# Dynamics in Deep Classifiers Trained with the Square Loss: Normalization, Low Rank, Neural Collapse, and Generalization Bounds

**DOI:** 10.34133/research.0024

**Published:** 2023-03-08

**Authors:** Mengjia Xu, Akshay Rangamani, Qianli Liao, Tomer Galanti, Tomaso Poggio

**Affiliations:** ^1^ Center for Brains, Minds and Machines, Massachusetts Institute of Technology, Cambridge, MA, USA.; ^2^ Division of Applied Mathematics, Brown University, Providence, RI, USA.

## Abstract

We overview several properties—old and new—of training overparameterized deep networks under the square loss. We first consider a model of the dynamics of gradient flow under the square loss in deep homogeneous rectified linear unit networks. We study the convergence to a solution with the absolute minimum *ρ*, which is the product of the Frobenius norms of each layer weight matrix, when normalization by Lagrange multipliers is used together with weight decay under different forms of gradient descent. A main property of the minimizers that bound their expected error for a specific network architecture is *ρ*. In particular, we derive novel norm-based bounds for convolutional layers that are orders of magnitude better than classical bounds for dense networks. Next, we prove that quasi-interpolating solutions obtained by stochastic gradient descent in the presence of weight decay have a bias toward low-rank weight matrices, which should improve generalization. The same analysis predicts the existence of an inherent stochastic gradient descent noise for deep networks. In both cases, we verify our predictions experimentally. We then predict neural collapse and its properties without any specific assumption—unlike other published proofs. Our analysis supports the idea that the advantage of deep networks relative to other classifiers is greater for problems that are appropriate for sparse deep architectures such as convolutional neural networks. The reason is that compositionally sparse target functions can be approximated well by “sparse” deep networks without incurring in the curse of dimensionality.

## Introduction

A widely held belief in the last few years has been that the cross-entropy loss is superior to the square loss when training deep networks for classification problems. As such, the attempts at understanding the theory of deep learning have been largely focused on exponential-type losses [1,2], such as the cross-entropy. For these losses, the predictive ability of deep networks depends on the implicit complexity control of gradient descent (GD) algorithms that lead to asymptotic maximization of the classification margin on the training set [1,3,4]. Recently, however, Hui and Belkin [5] have empirically demonstrated that it is possible to achieve a similar level of performance, if not better, using the square loss, paralleling older results for support vector machines [6]. Can a theoretical analysis explain when and why regression should work well for classification? This question was the original motivation for this paper and preliminary versions of it [7,8].

In deep learning binary classification, unlike the case of linear networks, we expect from previous results (in the absence of regularization) several global minima with zero square loss, thus corresponding to interpolating solutions (in general degenerate, see [9,10] and reference therein), because of overparametrization. Although all the interpolating solutions are optimal solutions to the regression problem, they will generally correspond to different (normalized) margins and to different expected classification performances. In other words, zero square loss does not imply by itself neither large margin nor good classification on a test set. When can we expect the solutions to the regression problem obtained by GD to have a large margin?

We introduce a simplified model of the training procedure that uses square loss, binary classification, gradient flow (GF), and Lagrange multipliers (LMs) for normalizing the weights. With this model, we show that obtaining large margin interpolating solutions depends on the scale of initialization of the weights close to zero, in the absence of regularization [also called weight decay (WD)]. Assuming convergence, we describe the qualitative dynamics of the deep network’s parameters and show that *ρ*, which is the product of the Frobenius norms of the weight matrices, grows nonmonotonically until a large margin, which is small *ρ* solution, is found reached. Assuming that local minima and saddle points can be avoided, this analysis suggests that with WD (or sometimes with just small initialization), GD techniques may yield convergence to a minimum with a *ρ* biased to be small.

In the presence of WD, perfect interpolation of all data points cannot occur and is replaced by quasi-interpolation of the labels. In the special case of binary classification case in which *y_n_* = ±1, quasi-interpolation is defined as ∀ *n*:|*f*(*x_n_*) − *y_n_* | ≤ *ϵ*, where *ϵ* > 0 is small. Our experiments and analysis of the dynamics show that in the presence of regularization, there is a weaker dependence on initial conditions, as has been observed in [5]. We show that WD helps stabilize normalization of the weights, in addition to its role in the dynamics of the norm.

We then apply basic bounds on expected error to the solutions provided by stochastic gradient descent (SGD) (for WD *λ* > 0), which have locally minimum *ρ*. For normal training set sizes, the bounds are still vacuous but much closer to the test error than previous estimates. This is encouraging because in our setup, large overparametrization, corresponding to interpolation of the training data [11], coexists with a relatively small Rademacher complexity because of the sparsity induced by the locality of the convolutional kernel. [By several orders of magnitude.]

We then turn to show that the quasi-interpolating solutions satisfy the recently discovered neural collapse (NC) phenomenon [12], assuming SGD with minibatches. According to NC, a dramatic simplification of deep network dynamics takes place—not only do all the margins become very similar to each other, but the last layer classifiers and the penultimate layer features also form the geometrical structure of a simplex equiangular tight frame (ETF). Here, we prove the emergence of NC for the square loss for the networks that we study—without any additional assumption (such as unconstrained features).

Finally, the study of SGD reveals surprising differences relative to GD. In particular, in the presence of regularization, SGD does not converge to a perfect equilibrium: There is always, at least generically, SGD noise. The underlying reason is a rank constraint that depends on the size of the minibatches. This also implies an SGD bias toward low-rank solutions that reinforces a similar bias due to maximization of the margin under normalization (which can be inferred from [13]).

### Contributions

The main original contributions in this paper are as follows:

• We analyze the dynamics of deep network parameters, their norm, and the margins under GF on the square loss, using Lagrange normalization (LN). We describe the evolution of *ρ* and the role of WD and normalization in the training dynamics. The analysis in terms of the “polar” coordinates *ρ*, *V_k_* is new, and many of the observed properties are not. Arguably, our analysis of the bias toward minimum *ρ* and its dynamics with and without WD is an original contribution.

• Our norm-based generalization bounds for convolutional neural networks (CNNs) are new. We outline in this paper a derivation for the case of nonoverlapping convolutional patches. The extension to the general case follows naturally and will be described in a forthcoming paper. The bounds show that generalization for CNNs can be orders of magnitude better than that for dense networks. In the experiments that we describe, the bounds turn out to be loose but close to nonvacuous. They appear to be much better than the other empirical tests of generalization bounds—all for dense networks—that we know of. The main reason for this, in addition to the relatively simple task (binary classification in CIAFR10), is the sparsity of the convolutional network, which is the low dimensionality (or locality) of the kernel.

• We prove that convergence of GD optimization with WD and normalization yields NC for deep networks trained with square loss in the binary and in the multiclass classification case. Experiments verify the predictions. Our proof is free of any assumption—unlike other recent papers that depend on the “unconstrained feature assumption”.

• We prove that training the network using SGD with WD induces a bias toward low-rank weight matrices. As we will describe in a separate paper, low rank can yield better generalization bounds.

• The same theoretical observation that predicts a low-rank-bias also predicts the existence of an intrinsic SGD noise in the weight matrices and in the margins.

## Related Work

There has been much recent work on the analysis of deep networks and linear models trained using exponential-type losses for classification. The implicit bias of GD toward margin maximizing solutions under exponential-type losses was shown for linear models with separable data in [14] and for deep networks in [1,2,15,16]. Recent interest in using the square loss for classification has been spurred by the experiments in [5], although the practice of using the square loss is much older [6]. Muthukumar et al. [17] recently showed for linear models that interpolating solutions for the square loss are equivalent to the solutions to the hard margin support vector machine problem (see also [7]). Recent work also studied interpolating kernel machines [18,19] that use the square loss for classification.

In the recent past, there have been a number of papers analyzing deep networks trained with the square loss. These include the works of Zhong et al. [20] and Soltanolkotabi et al. [21] that show how to recover the parameters of a neural network by training on data sampled from it. The square loss has also been used in analyzing convergence of training in the neural tangent kernel (NTK) regime [22–24]. Detailed analyses of 2-layer neural networks such as [25–27] typically use the square loss as an objective function. However, these papers do not specifically consider the task of classification.

A large effort has been spent in understanding generalization in deep networks. The main focus has been solving the puzzle of how overparameterized deep networks (with more parameters than data) are able to generalize. An influential paper [11] showed that overparameterized deep networks that usually fit randomly labeled data also generalize well when they trained on correctly labeled data. Thus, the training error does not give any information about test error: There is no uniform convergence of training error to test error. This is related to another property of overparametrization: Standard Vapnik–Chervonenkis bounds are always vacuous when the number of parameters is larger than the number of data. Although often forgotten, it is, however, well known that another type of bounds—on the norm of parameters—may provide generalization even if there are more parameters than data. This point was made convincingly in [28], which provides norm-based bounds for deep networks. [The focus of this paper on *ρ* is directly related.] Bartlett bounds and related ones [29,30] in practice turn out to be very loose. Empirical studies such as [31] found little evidence so far that norms and margins correlate well with generalization.

NC [12] is a recently discovered empirical phenomenon that occurs when training deep classifiers using the cross-entropy loss. Since its discovery, there have been a few papers analytically proving its emergence when training deep networks. Mixon et al. [32] show NC in the regime of “unconstrained features”. Recent results in [33] perform a more comprehensive analysis of NC in the unconstrained features paradigm. There have been a series of papers analytically showing the emergence of NC when using the cross-entropy loss [34–36]. In the study of the emergence of NC when training using the square loss, Ergen and Pilanci [37] (see also [38]) derived it through a convex dual formulation of deep networks. In addition to that, Han et al. [39] and Zhou et al. [40] show the emergence of NC in the unconstrained features regime. Our independent derivation is different from these approaches and shows that NC emerges in the presence of normalization and WD.

Several papers in recent years have studied the relationship between implicit regularization in linear neural networks and rank minimization. A main focus was on the matrix factorization problem, which corresponds to training a depth-2 linear neural network with multiple outputs with respect to the square loss (see references in [13]). Beyond factorization problems, it was shown that in linear networks of output dimension 1, GF with respect to exponential-type loss functions converges to networks where the weight matrix of every layer is of rank 1. However, for nonlinear neural networks, things are less clear. Empirically, several studies (see references in [13]) showed that replacing the weight matrices by low-rank approximations results in only a small drop in accuracy. This suggests that the weight matrices in practice are not too far from being low rank.

## Problem Setup

In this section, we describe the training settings considered in our work. We study training deep neural network with rectified linear unit (ReLU) nonlinearity using square loss minimization for classification problems. In the proposed analysis, we apply a specific normalization technique: weight normalization (WN), which is equivalent to LM, and regularization (also called WD), since such mechanisms seem commonly used for reliably training deep networks using GD techniques [5,41].

### Assumptions

Throughout the theoretical analysis, we make, in some places, simplifying assumptions relative to standard practice in deep neural networks. We mostly consider that the case of binary classification though our analysis of NC includes multiclass classification. We restrict ourselves to the square loss. We consider GD techniques, but we assume different forms of them in various sections of the paper. In the first part, we assume continuous GF instead of GD or SGD. GF is the limit of discrete GD algorithm with the learning rate being infinitesimally small (we describe an approximation of GD within a GF approach in [8]). SGD is specifically considered and shown to bias rank and induce asymptotic noise that is unique to it. The analysis of NC is carried out using SGD with small learning rates. Furthermore, we assume WN by an LM term added to the loss function, which normalizes the weight matrices. This is equivalent to WN but is not equivalent to the more commonly used batch normalization (BN).

We also assume throughout that the network is overparameterized and so that there is convergence to global minima with appropriate initialization, parameter values, and data.

### Classification with square loss minimization

**Fig. 1.**
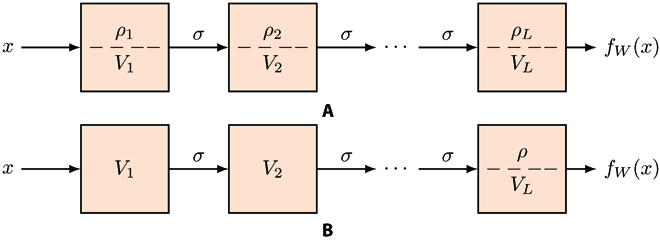
An illustration of 2 parametrizations of *f_W_*(*x*). In (A), we decompose each layer’s weight matrix *W_i_* into its norm *ρ_i_* and its normalized version *V_i_*. In (B), we normalize each layer except for the top layer’s matrix *W_L_* that is decomposed into a global *ρ* and the last layer *V_L_*. Normalizing the weight matrices, as WN (equivalent to LN) does, is different from BN, although both normalization techniques capture the relevant property of normalization—to make the dot product invariant to scale.

In this work, we consider a square loss minimization for classification along with regularization and WN. We consider a binary classification problem, given a training dataset $S={\left\{\left(x_{n},y_{n}\right)\right\}}_{n=1}^{N}$, where *x_n_* ∈ ℝ*^d^* is the input (normalized such that ∥*x_n_* ∥ ≤ 1) and *y_n_* ∈ {±1} is the label. We use deep rectified homogeneous networks with *L* layers to solve this problem. For simplicity, we consider networks *f_W_* : ℝ*^d^* → ℝ*^p^* of the following form *f_W_*(*x*) = *W_L_σ*(*W*_*L* − 1_…*σ*(*W*_1_*x*)…), where *x* ∈ ℝ*^d^* is the input to the network and *σ* : ℝ → ℝ, *σ*(*x*) = *max* (0, *x*) is the ReLU activation function that is applied coordinate-wise at each layer. The last layer of the network is linear (see Fig. 1).

Because of the positive homogeneity of ReLU [i.e., *σ*(*αx*) = *ασ*(*x*) for all *x* ∈ ℝ and *α* > 0], one can reparametrize *f_W_*(*x*) by considering normalized weight matrices $V_{k}=\frac{W_{k}}{∥W_{k}∥}$ and define *ρ_k_* = ∥ *W_k_*∥, obtaining *f_W_*(*x*) = *ρ_L_V_L_σ*(*ρ*_*L* − 1_…*σ*(*ρ*_1_*V*_1_*x*)…). [We choose the Frobenius norm here.] Because of homogeneity of the ReLU, it is possible to pull out the product of the layer norms as *ρ* = ∏*_k_* *ρ_k_* and write *f_W_*(*x*) = *ρf_V_*(*x*) = *ρV_L_σ*(*V*_*L* − 1_…*σ*(*V*_1_*x*)…). Notice that the 2 networks—*f_W_*(*x*) and *ρf_V_*(*x*)—are equivalent reparameterizations of the same function (if *ρ* = ∏*_k_* *ρ_k_*) but their optimization generally differ. We define *f_n_* ≔ *f_V_*(*x_n_*).

We adopt in our definition the convention that the norm *ρ_j_* of the convolutional layers is the norm of their filters and not the norm of their associated Toeplitz matrices. The reason is that this is what our novel bounds for CNNs state (see also section 3.3 in [42,43]). The total *ρ* calculated in this way is the quantity that enters the generalization bounds of Generalization: Rademacher Complexity of Convolutional Layers.

In practice, certain normalization techniques are used to train neural networks. This is usually performed using either BN or, less frequently, WN. BN consists of standardizing the output of the units in each layer to have zero mean and unit variance with respect to training set. WN normalizes the weight matrices (section 10 in [4]). In our analysis, we model normalization by normalizing the weight matrices, using an LM term added to the loss function. This approach is equivalent to WN.

In the presence of normalization, we assume that all layers are normalized, except for the last one, via the added LM. Thus, the weight matrices ${\left\{V_{k}\right\}}_{k=1}^{L}$ are constrained by the LM term to be close to, and eventually converge to, unit norm matrices (in fact, to fixed norm matrices); notice that normalizing *V_L_* and then multiplying the output by *ρ* are equivalent to letting *W_L_* = *ρV_L_* be unnormalized. Thus, *f_V_* is the network that, at convergence, has *L* − 1 normalized layers (see Fig. 1B).

We can write the Lagrangian corresponding to the minimization of the regularized loss function under the constraint ∥*V_k_*∥^2^ = 1 in the following manner:

$$\begin{array}{cc} L_{S}\left(\rho ,{\left\{V_{k}\right\}}_{k=1}^{L}\right): & =\frac{1}{N}\sum_{n}{\left(\rho f_{n}-y_{n}\right)}^{2}+\sum_{k=1}^{L}\nu_{k}\left(∥V_{k}{∥}^{2}-1\right)+\lambda \rho^{2}\\ & =\frac{1}{N}\sum_{n}{\left(1-\rho {\bar{f}}_{n}\right)}^{2}+\sum_{k=1}^{L}\nu_{k}\left(∥V_{k}{∥}^{2}-1\right)+\lambda \rho^{2} \end{array}$$ (1)

where *ν_k_* values are the LMs and *λ* > 0 is a predefined parameter.

#### Separability and margins

Two important aspects of classification are separability and margins. For a given sample (*x*, *y*) (train or test sample) and model *f_W_*, we say that *f_W_* correctly classifies *x*, if ${\bar{f}}_{n}=y_{n}f_{n}>0$. In addition, for a given dataset $S={\left\{\left(x_{n},y_{n}\right)\right\}}_{n=1}^{N}$, separability is defined as the condition in which all training samples are classified correctly, $\forall n\in \left[N\right]:{\bar{f}}_{n}>0$. Furthermore, when $\sum_{n=1}^{N}{\bar{f}}_{n}>0$, we say that average separability is satisfied. The minimum of $L_{S}$ for *λ* = 0 is usually zero under our assumption of overparametrization. This corresponds to separability.

Notice that if *f_W_* is a zero loss solution of the regression problem, then ∀*n* : *f_W_*(*x_n_*) = *y_n_*, which is also equivalent to *ρf_n_* = *y_n_*, where we call $y_{n}f_{n}={\bar{f}}_{n}$ the margin for *x_n_*. By multiplying both sides of this equation by *y_n_* and summing both sides over *n* ∈ [*N*], we obtain that $\rho \sum_{n}{\bar{f}}_{n}=N$. Thus, the norm *ρ* of a minimizer is inversely proportional to its average margin *μ* in the limit of *λ* = 0, with $\mu =\frac{1}{N}\sum_{n}{\bar{f}}_{n}$. It is also useful to define the margin variance *σ*^2^ = *M* − *μ*^2^ with $M=\frac{1}{N}\sum_{n}{\bar{f}}_{n}^{2}$. Notice that $M=\frac{1}{N}\sum_{n}{\bar{f}}_{n}^{2}=\sigma^{2}+\mu^{2}$ and that both *M* and *σ*^2^ are not negative. [Notice that the term “margin” is usually defined as $\min_{n\in \left[N\right]}{\bar{f}}_{n}$. Instead, we use the term “margin for *x_n_*” to distinguish our definition from the usual one.]

#### Interpolation and quasi-interpolation

Assume that the weights *V_k_* are normalized at convergence. Then

Lemma 1. For *λ* = 0, there are solutions that interpolate all data points with the same margin and achieve zero loss. For λ > 0, there are no solutions that have the same margins and interpolate. However, there are solutions with the same margins that quasi-interpolate and are critical points of the gradient.

*Proof*. Consider the loss $L_{S}=\frac{1}{N}\sum_{n}{\left(1-\rho {\bar{f}}_{n}\right)}^{2}+\lambda \rho^{2}=1-2\rho \mu +\rho^{2}M+\lambda \rho^{2}$. For *λ* = 0, a zero of the loss $L_{S}=0$ implies $\forall n\in \left[N\right]:\mu ={\bar{f}}_{n}$ and $\mu =\frac{1}{\rho }$. However, for *λ* > 0, the assumption that all ${\bar{f}}_{n}$ values are equal yields *M* = *μ*^2^ and, thus, $L_{S}=\rho^{2}\mu^{2}-2\rho \mu +\left(1+\lambda \rho^{2}\right)$. Setting $L_{S}=0$ gives a second-order equation in *ρ* that does not have real-valued solutions for *λ* > 0. Thus, in the presence of regularization, there exist no solutions that have the same margin for all points and reach zero empirical loss. However, solutions that have the same margin for all points and correspond to zero gradient with respect to *ρ* exist. To see this, assume *σ* = 0, setting the gradient of $L_{S}$ with respect to *ρ* equal to zero, yielding *ρμ*^2^ − *μ* + *λρ* = 0. This gives $\rho =\frac{\mu }{\mu^{2}+\lambda }$. This solution yields *ρμ* < 1, which corresponds to noninterpolating solutions.

The Neural collapse section shows that the margins [which are never interpolating; interpolation is equivalent to *ρy_n_f*(*x_n_*) = 1] tend to become equal to each other as predicted from the lemma during convergence.

#### Experiments

We performed binary classification experiments using the standard CIFAR10 dataset [44]. Image samples with class labels 1 and 2 were extracted for the binary classification task. A total number of training and test data points are 10,000 and 2,000, respectively. The model architecture in Fig. 1B contains 4 convolutional layers and 2 fully connected layers with hidden sizes of 1,024 and 2. A number of channels for the 4 convolutional layers are 32, 64, 128, and 128, and the filter size is 3 × 3. The first fully connected layer has 3,200 × 1,024 = 3,276,800 weights, and the very last layer has 1,024 × 2 = 2,048 weights. At the top layer of our model, there is a learnable parameter *ρ* (Fig. 1B). In our experiments, instead of using LMs, we used the equivalent (see proof of the equivalence [2]) WN algorithm, freezing the weights of the WN parameter “g” [45] and normalizing the ${\left\{V_{k}\right\}}_{k=1}^{L-1}$ matrices at each layer with respect to their Frobenius norm, while the top layer’s norm is denoted by *ρ* and is the only parameter entering in the regularization term (see Eq. 11).

### Landscape of the empirical risk

**Fig. 2.**
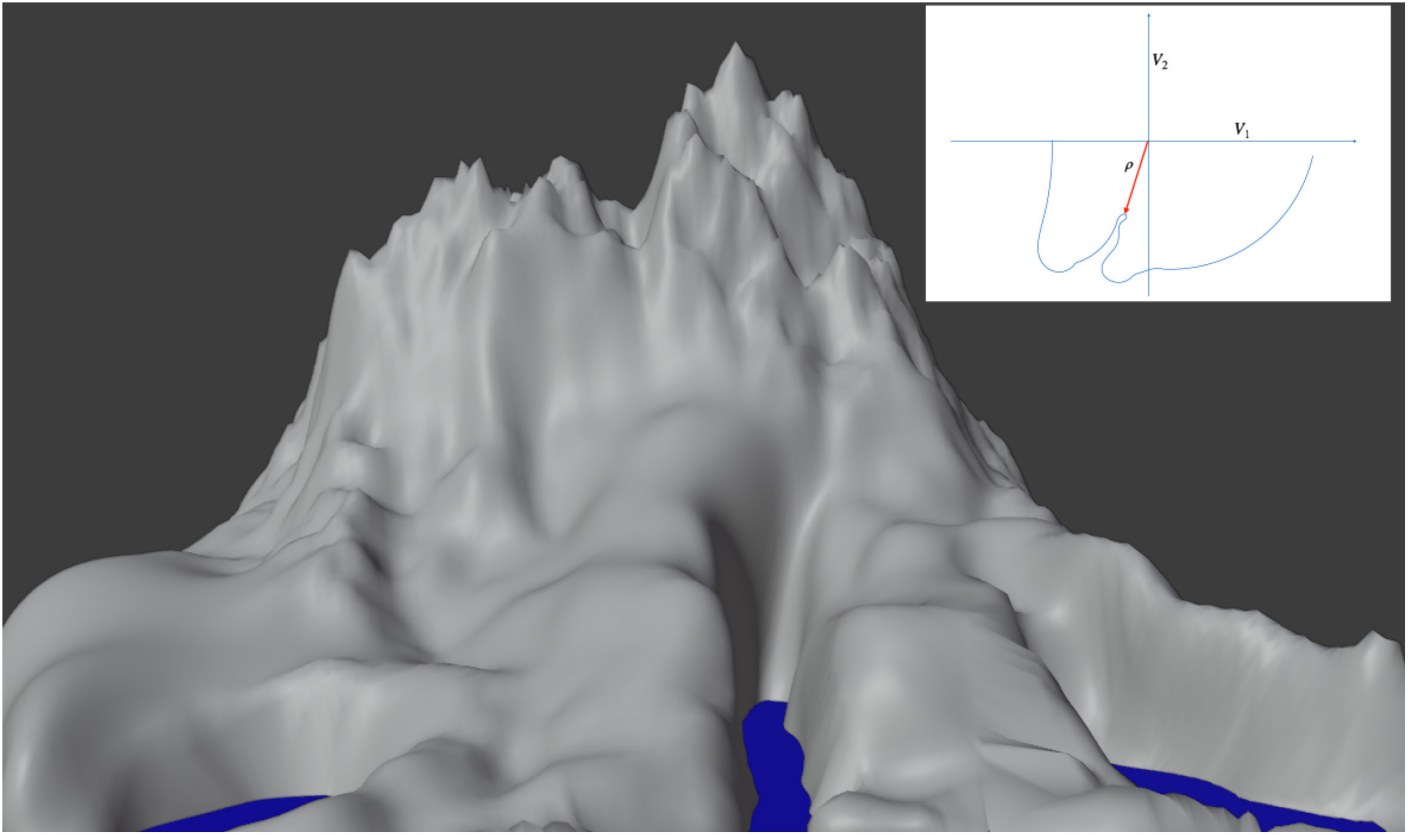
A speculative view of the landscape of the unregularized loss—which is for *λ* = 0. Think of the loss as the mountain emerging from the water with zero loss being the water level. *ρ* is the radial distance from the center of the mountain as shown in the inset, whereas the *V_k_* can be thought as multidimensional angles in this “polar” coordinate system. There are global degenerate valleys for *ρ* ≥ *ρ*_0_ with *V*_1_ and *V*_2_ weights of unit norm. The coastline of the loss marks the boundary of the zero-loss degenerate minimum where L=0 in the high-dimensional space of *ρ* and *V_k_* ∀ *k* = 1, ⋯, *L*. The degenerate global minimum is shown here as a connected valley outside the coastline. The red arrow marks the minimum loss with minimum *ρ*. Notice that, depending on the shape of the multidimensional valley, regularization with a term *λρ*^2^ added to the loss biases the solution toward small *ρ* but does not guarantee convergence to the minimum *ρ* solution, unlike the case of a linear network.

As a next step, we establish key properties of the loss landscape. The landscape of the empirical loss contains a set of degenerate zero-loss global minima (for *λ* = 0) that under certain overparametrization, assumptions may be connected in a single zero-loss degenerate valley for *ρ* ≥ *ρ*_0_. Figure 2 shows a landscape that has a saddle for *ρ* = 0 and then goes to zero loss (zero crossing level, that is the coastline) for different values of *ρ* (look at the boundary of the mountain). As we will see in our analysis of the GF, the descent from *ρ* = 0 can encounter local minima and saddles with nonzero loss. Furthermore, although the valley of zero loss may be connected, the point of absolute minimum *ρ* may be unreachable by GF from another point of zero loss even in the presence of *λ* > 0, because of the possible nonconvex profile of the coastline (see inset of Fig. 2).

If we assume overparameterized networks with *d* ≫ *n*, where *d* is the number of parameters and *N* is the number of data points, the study of Cooper [10] proved that the global minima of the unregularized loss function $L_{S}=\sum_{i=1}^{N}{\left(f_{W}\left(x_{i}\right)-y_{i}\right)}^{2}$ are highly degenerate with dimension *d* − *N*. [This result is also what one expects from Bezout theorem for a deep polynomial network. As mentioned in T. Tao’s blog “from the general “soft” theory of algebraic geometry, we know that the algebraic set *V* is a union of finitely many algebraic varieties, each of dimension at least *d − N*, with none of these components contained in any other. In particular, in the underdetermined case *N* < *d*, there are no zero-dimensional components of *V* , and, thus, *V* is either empty or infinite”(see references in [46]).]

Theorem 1 ([46], informal)**.** We assume an overparameterized neural network *f_W_* with smooth ReLU activation functions and square loss. Then, the minimizers *W*^∗^ achieve zero loss and are highly degenerate with dimension *d* − *N*.

Furthermore, for “large” overparametrization, all the global minima—associated with interpolating solutions—are connected within a unique, large valley. The argument is based on Theorem 5.1 of [47]:

Theorem 2 ([47], informal)**.** If the first layer of the network has at least 2*N* neurons, where *N* is the number of training data, and if the number of neurons in each subsequent layer decreases, then every sublevel set of the loss is connected.

In particular, the theorem implies that zero-square-loss minima with different values of ρ are connected. A connected single valley of zero loss does not, however, guarantee that SGD with WD will converge to the global minimum, which is now >0, independently of initial conditions.

For large *ρ*, we expect many solutions. The existence of several solutions for large *ρ* is based on the following intuition: The last linear layer is enough—if the layer before the linear classifier has more units than the number of training points—to provide solutions for a given set of random weights in the previous layers (for large *ρ* and small *f_i_*). This also means that the intermediate layers do not need to change much under GD in the iterations immediately after initialization. The emerging picture is a landscape in which there are no zero-loss minima for *ρ* smaller than a certain minimum *ρ*, which is network and data dependent. With increasing *ρ* from *ρ* = 0, there will be a continuous set of zero-square-loss degenerate minima with the minimizer representing an interpolating (for *λ* = 0) or almost interpolating solution (for *λ* > 0). We expect that *λ* > 0 results in a “pull” toward the minimum *ρ*_0_ within the local degenerate minimum of the loss.

#### *Landscape for* *λ >* 0

In the case of *λρ*^2^ > 0, the landscape may become a Morse–Bott or Morse function with shallow almost zero-loss minima. The question is open because the regularizer is not the sum of squares.

### Gradient dynamics

#### GF equations

The GF equations are as follows (see also [8]):

$$\begin{array}{c} \overset{\cdot }{\rho }=-\frac{\partial L_{S}\left(\rho ,{\left\{V_{k}\right\}}_{k=1}^{L}\right)}{\partial \rho }=\frac{2}{N}\sum_{n}\left(1-\rho {\bar{f}}_{n}\right){\bar{f}}_{n}-2\lambda \rho ,\\ {\overset{\cdot }{V}}_{k}=-\frac{\partial L_{S}\left(\rho ,{\left\{V_{k}\right\}}_{k=1}^{L}\right)}{\partial V_{k}}=\frac{2}{N}\sum_{n}\left(1-\rho {\bar{f}}_{n}\right)\rho \frac{\partial {\bar{f}}_{n}}{\partial V_{k}}-2\nu_{k}V_{k} \end{array}$$ (2)

In the second equation, we can use the unit norm constraint on the ∥*V_k_*∥ to determine the LMs *ν_k_*, using the following structural property of the gradient:

Lemma 2 (Lemma 2.1 of [48])**.** Let f_W_(*x*) be a ReLU neural network, *f_W_*(*x*) = *W_L_σ*(*W*_*L* − 1_…*σ*(*W*_1_*x*)) : ℝ*^d^* → ℝ.* Then, we can write:*

$$\forall x\in {\mathbb{R}}^{d}:\sum_{i,j}\frac{\partial f_{W}\left(x\right)}{\partial W_{k}^{i,j}}W_{k}^{i,j}=\langle W_{k},\frac{\partial f_{W}\left(x\right)}{\partial W_{k}}\rangle =f_{W}\left(x\right)$$ (3)

The constraint ∥*V_k_*∥^2^ = 1 implies using the lemma above $\frac{\partial ∥V_{k}{∥}^{2}}{\partial t}=V_{k}^{T}{\overset{\cdot }{V}}_{k}=0$, which gives

$$\nu_{k}=\frac{1}{N}\sum_{n}\left(\rho {\bar{f}}_{n}-\rho^{2}f_{n}^{2}\right)=\frac{1}{N}\sum_{n}\rho {\bar{f}}_{n}\left(1-\rho f_{n}\right)$$ (4)

Thus, the GF is the following dynamical system

$$\overset{\cdot }{\rho }=\frac{2}{N}\left[\sum_{n}{\bar{f}}_{n}-\sum_{n}\rho {\left({\bar{f}}_{n}\right)}^{2}\right]-2\lambda \rho \;\text{and}\;{\overset{\cdot }{V}}_{k}=\frac{2}{N}\rho \sum_{n}\left[\left(1-\rho {\bar{f}}_{n}\right)\left(-V_{k}{\bar{f}}_{n}+\frac{\partial {\bar{f}}_{n}}{\partial V_{k}}\right)\right]$$ (5)

In particular, we can also write

$$\overset{\cdot }{\rho }=2\left(\mu -\rho \left(M+\lambda \right)\right)$$ (6)

Hence, at critical points (when $\overset{\cdot }{\rho }=0$ and ${\overset{\cdot }{V}}_{k}=0$), we used the definitions of *μ* and *M*,

$$\rho =\rho_{\mathrm{eq}}≔\frac{\frac{1}{N}\sum_{n}{\bar{f}}_{n}}{\lambda +\frac{1}{N}\sum_{n}{\bar{f}}_{n}^{2}}=\frac{\mu }{M+\lambda }$$ (7)

Thus, the gap to interpolation due to *λ* > 0 is $\epsilon =\left(\rho_{\lambda =0}-\rho_{\lambda }\right)\mu =1-\frac{\mu }{M+\lambda }\mu$ that gives

$$\epsilon =1-\frac{\mu^{2}}{\mu^{2}+\sigma^{2}+\lambda }=\frac{\sigma^{2}+\lambda }{\mu^{2}+\sigma^{2}+\lambda }$$ (8)

Notice that since the *V_k_* values are bounded functions, they must take their maximum and minimum values on their compact domain—the sphere—because of the extremum value theorem. In addition, notice that for normalized *V_k_*, $V_{k}^{T}{\overset{\cdot }{V}}_{k}=0$ always that is *V_k_* can only rotate. If ${\overset{\cdot }{V}}_{k}=0$, then the weights *V_k_* are given by

$$V_{k}=\frac{\sum_{n}\ell_{n}\frac{\partial f_{n}}{\partial V_{k}}}{\sum \ell_{n}f_{n}}$$ (9)

where $\ell_{n}=1-\rho {\bar{f}}_{n}$. [This overdetermined system of equations—with as many equations as weights—can also be used to reconstruct the training set from the *V_k_*, the *y_n_*, and the *f_n_*.]

#### Convergence

A favorable property of optimization of the square loss is the convergence of the relevant parameters. With GD, the loss function cannot increase, while the trainable parameters may potentially diverge. A typical scenario of this kind happens with cross-entropy minimization, where the weights typically tend to infinity. In light of the theorems in the Landscape of the empirical risk section, we could hypothetically think of training dynamics in which the loss function’s value $L\left(\rho ,{\left\{V_{k}\right\}}_{k=1}^{L}\right)$ decreases, while *ρ* oscillates periodically within some interval. As we show next, this is impossible when the loss function’s value converges to zero.

Lemma 3. Let f_W_(*x*) = *ρf_V_*(*x*) be a neural network and λ = 0. Assume that during training time, we have $\lim_{t\to \infty }L\left(\rho ,{\left\{V_{k}\right\}}_{k=1}^{L}\right)=0$ and ∀*k* ∈ [*L*] : ∥ *V_k_* ∥ = 1. Then, *ρ* and *V_k_* converge (i.e., $\overset{\cdot }{\rho }\to 0$ and ${\overset{\cdot }{V}}_{k}\to 0$*).*

*Proof.* Note that if $\lim_{t\to \infty }L\left(\rho ,{\left\{V_{k}\right\}}_{k=1}^{L}\right)=0$, then, for all *n* ∈ [*N*], we have (*ρf_n_* − *y_n_*)^2^ → 0. In particular, *ρf_n_* → *y_n_* and $\rho {\bar{f}}_{n}\to 1$. Hence, we conclude that *μρ* → 1. Therefore, by Lemma 4, $\rho \overset{\cdot }{\rho }\to 0$. We note that *ρ* → 0 would imply *ρf_n_* → 0 that contradicts $L\left(\rho ,{\left\{V_{k}\right\}}_{k=1}^{L}\right)\to 0$, since the labels *y_n_* are nonzero. Therefore, we conclude that $\overset{\cdot }{\rho }\to 0$. To see why ${\overset{\cdot }{V}}_{k}\to 0$, we recall that

$${\overset{\cdot }{V}}_{k}=\frac{2}{N}\rho \sum_{n}\left[\left(1-\rho {\bar{f}}_{n}\right)\left(-V_{k}{\bar{f}}_{n}+\frac{\partial {\bar{f}}_{n}}{\partial V_{k}}\right)\right]$$ (10)

We note that ∥*V_k_* ∥ = 1, $|{\bar{f}}_{n}|=1$, and $\frac{\partial {\bar{f}}_{n}}{\partial V_{k}}$ is bounded (assuming that ∀*n* ∈ [*N*] : ∥ *x_n_* ∥ ≤ 1 and ∀*k* ∈ [*L*] : ∥ *V_k_* ∥ = 1). Hence, since *ρ* converges, $\rho {\bar{f}}_{n}\to 1$, implying (for *λ* = 0) ${\overset{\cdot }{V}}_{k}\to 0$.

So far, we have assumed convergence of GF, GD, or SGD to zero loss. Convergence does not seem too far-fetched given overparametrization and the associated high degeneracy of the global minima (see Landscape of the empirical risk section and theorems there). Proofs of convergence of descent methods have been, however, lacking until a recent paper [49] presented a new criterion for convergence of GD and used to show that GD with proper initialization converges to a global minimum. The result has technical limitations that are likely to be lifted in the future: It assumes that the activation function is smooth, that the input dimension is greater than or equal to the number of data points, and that the descent method is GF or GD.

### Qualitative dynamics

We consider the dynamics of model in Fig. 1B. During training the norm of each layer, weight matrix is kept constant by the LM constraint that is applied to all layers but the last one, thus leaving *ρ* at the top to change depending on the dynamics. Recall that $\forall n\in \left[N\right]:0\leq |{\bar{f}}_{n}|\leq 1$ because the assumption ∥*x* ∥ ≤ 1 yields ∥*f*(*x*) ∥ ≤ 1 by taking into account the definition of ReLUs and the fact that matrix norms are submultiplicative. Depending on the number of layers, the maximum margin that the network can achieve for a given dataset is usually much smaller than the upper bound 1, because the weight matrices have unit norm and the bound ≤1 is conservative. Thus, to guarantee interpolation, namely, *ρf_n_y_n_* = 1, *ρ* must be substantially larger than 1. For instance, in the experiments plotted in this paper, the maximal ${\bar{f}}_{n}$ is ≈0.002, and, thus, the *ρ* needed for interpolation (for *λ* = 0) is in the order of 500. We assume then that for a given dataset, there is a maximal value of *y_n_f_n_* that allows interpolation. Correspondingly, there is a minimum value of *ρ* that we call, as mentioned earlier, *ρ*_0_.

We now provide some intuition for the dynamics of the model. Notice that *ρ*(*t*) = 0 and *f_V_*(*x*) = 0 (if all weights are zero) are critical unstable points. A small perturbation will either result in $\overset{\cdot }{\rho }<0$ with *ρ* going back to zero or in *ρ* growing if the average margin is just positive, that is, *μ* > *λρ* > 0.

#### Small ρ initialization

First, we consider the case where the neural network is initialized with a smallish *ρ*, that is, *ρ* < *ρ*_0_. Assume then that at some time *t*, *μ* > 0, that is, average separability holds. Notice that if the *f_n_* values were zero-mean, random variables, then there would be a 50% chance for average separability to hold. Then, Eq. 5 shows that $\overset{\cdot }{\rho }>0$. If full separability takes place, that is, ∀*n* : *f_n_* > 0, then $\overset{\cdot }{\rho }$ remains positive at least until *ρ* = 1. This is because Eq. 5 implies that $\overset{\cdot }{\rho }\geq 2\left(\mu -\rho \mu \right)$ since *M* ≤ *μ*. In general, assuming eventual convergence, *ρ* may grow nonmonotonically, that is, there may oscillations in *ρ* for “short” intervals, until it converges to *ρ*_0_.

To see this, consider the following lemma that gives a representation of the loss function in terms of *ρ*, $\overset{\cdot }{\rho }$, and *μ*.

Lemma 4. *Let f_W_*(*x*) = *ρf_V_*(*x*) be a neural network, with ∀*k* ∈ [*L*] : ∥ *V_k_* ∥ = 1. The square loss can be written as $L_{S}\left(\rho ,{\left\{V_{k}\right\}}_{k=1}^{L}\right)=1-\rho \left(\frac{1}{2}\overset{\cdot }{\rho }+\mu \right)$.

*Proof*. First, we consider that

$$\begin{array}{cc} L_{S}\left(\rho ,{\left\{V_{k}\right\}}_{k=1}^{L}\right) & =\frac{1}{N}\sum_{n}{\left(\rho f_{n}-y_{n}\right)}^{2}+\sum_{k=1}^{L}\nu_{k}\left(∥V_{k}{∥}^{2}-1\right)+\lambda \rho^{2}\\ & =\frac{1}{N}\left(\rho^{2}f_{n}^{2}-2y_{n}\rho f_{n}+y_{n}^{2}\right)+\lambda \rho^{2}\\ & =1-2\rho \mu +\rho^{2}M+\lambda \rho^{2} \end{array}$$ (11)

where the second equation follows from ∀*k* ∈ [*L*] : ∥ *V_k_* ∥ = 1 and the third equation follows from $y_{n}^{2}=1$, using the previous definitions $\mu =\frac{1}{N}\sum_{n}{\bar{f}}_{n}$ and $M=\frac{1}{N}\sum_{n}{\bar{f}}_{n}^{2}$. On the other hand, by Eq. 6, $\overset{\cdot }{\rho }=2\mu -2\rho M-2\lambda \rho$ that gives $2\rho M=2\mu -2\lambda \rho -\overset{\cdot }{\rho }$. Therefore, we conclude that $L_{S}\left(\rho ,{\left\{V_{k}\right\}}_{k=1}^{L}\right)=1-\frac{1}{2}\rho \overset{\cdot }{\rho }-\rho \mu =1-\rho \left(\frac{1}{2}\overset{\cdot }{\rho }+\mu \right)$ as desired.

Following this lemma, if $\overset{\cdot }{\rho }$ becomes negative during training, then the average margin *μ* must increase since GD cannot increase but only decrease L. In particular, this implies that $\overset{\cdot }{\rho }$ cannot be negative for long periods of time. Notice that short periods of decreasing *ρ* are “good” since they increase the average margin.

If $\overset{\cdot }{\rho }$ turns negative, then it means that it has crossed $\overset{\cdot }{\rho }=0$. This may be a critical point for the system if the values of *V_k_* corresponding to ${\overset{\cdot }{V}}_{k}=0$ are compatible (since the matrices ${\left\{V_{k}\right\}}_{k=1}^{L}$ determine the value of ${\bar{f}}_{n}$). We assume that this critical point—either a local minimum or a saddle—can be avoided by the randomness of SGD or by an algorithm that restarts optimization when a critical point is reached for which L>0.

Thus, *ρ* grows (nonmonotonically) until it reaches an equilibrium value, close to *ρ*_0_. Recall that for *λ* = 0, this corresponds to a degenerate global minimum L=0, usually resulting in a large attractive basin in the loss landscape. For *λ* = 0, a zero value of the loss (L=0) implies interpolation: Thus, all the *f_n_* have the same value, that is, all the margins are the same.

#### Large ρ initialization

If we initialize a network with large norm *ρ* > *ρ*_0_, then Eq. 1 shows that $\overset{\cdot }{\rho }<0$. This implies that the norm of the network will decrease until, eventually, an equilibrium is reached. In fact, since *ρ* ≫ 1, it is likely that there exists an interpolating (or near interpolating) solution with *ρ* that is very close to the initialization. In fact, for large *ρ*, it is usually empirically possible to find a set of weights *V_L_*, such that $\rho {\bar{f}}_{n}\approx 1$. To understand why this may be true, recall that if there are at least *N* units in the top layer of the network (layer *L*) with given activities and *ρ* ≫ *ρ*_0_, then there exist values of *V_L_* that yield interpolation due to Theorem 2. In other words, it is easy for the network to interpolate with small values ${\bar{f}}_{n}$. These large *ρ*, small ${\bar{f}}_{n}$ solutions are reminiscent of the NTK solutions [24], where the parameters do not move too far from their initialization. A formal version of the same argument is based on the following result.

We now assume that the network in the absence of WD has converged to an interpolating solution

Lemma 5. *Let f_V_ be a neural network with weights* ${\left\{V_{k}\right\}}_{k=1}^{L}$, such that, $\forall n\in \left[N\right]:\rho {\bar{f}}_{n}=\rho \mu^{*}=1$. Further assume that the classifier *V_L_ and the last layer features h are aligned, i.e., y_n_*〈*V_L_*, *h*(*x_n_*)〉 = ‖*h*(*x_n_*)‖_2_*, where the vector h denotes the activities of the units in the last layer. Then, perturbing V_L_ into another unit-norm vector $V_{L}^{\prime }\in {\mathbb{R}}^{p}$*, such that $V_{L}^{T}V_{L}^{\prime }=\alpha \in (0,1)$ yields a neural network $\hat{f}(x)=\langle V_{L}^{\prime },h(x)\rangle$ with the property that $\frac{\rho }{\alpha }\hat{f}$ is an interpolating solution, corresponding to a critical point of the gradient but with a larger *ρ*.

*Proof*. Consider the margins of the network $\hat{f}\left(x\right)=\langle V_{L}^{\prime },h(x)\rangle$. We conclude that ${\bar{\hat{f}}}_{n}=y_{n}\langle V_{L}^{\prime },h(x_{n})\rangle$. Since the classifier weights and the last layer features are aligned (as it may happen for *λ* → 0), we have that *y_n_h*(*x_n_*) = ‖*h*(*x_n_*)‖ × *V_L_*. This means ${\bar{\hat{f}}}_{n}=\|h(x_{n})\|\times \langle V_{L}^{\prime },V_{L}\rangle$. We also have from the interpolating condition that $\rho {\bar{f}}_{n}=\rho \mu^{*}=1$, which means $\|h\left(x_{n}\right)\|=\frac{1}{\rho }$. Putting all this together, we have $\frac{\rho }{\alpha }{\bar{\hat{f}}}_{n}=1$, which concludes the proof.

Thus, if a network exists providing an interpolating solution with a minimum *ρ* and *V_L_* ∝ *h*, there exist networks that differ only in the last *V_L_* layer and are also interpolating but with larger *ρ*. As a consequence, there is a continuum of solutions that differ only in the weights *V_L_* of the last layer.

Of course, there may be interpolating solutions corresponding to different sets of weights in layers below *L*, to which the above statement does not apply. These observations suggest that there is a valley of minimizers for increasing *ρ*, starting from a zero-loss minimizer that has the NC property (see Neural Collapse).

**Fig. 3.**
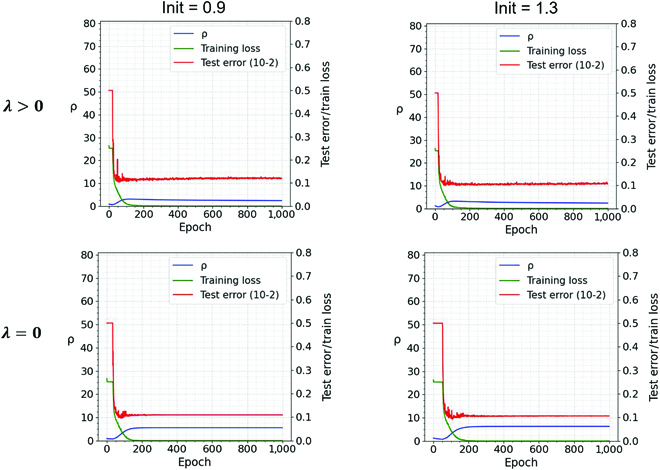
Training dynamics of *ρ* of the training loss and of the test error over 1,000 epochs with different initialization (0.9) in the first column and (1.3) in the second column. The number of channels for the 4 convolutional layers (Conv1 to Conv4) are 32, 64, 128, and 128, the filter size is 3 × 3, the hidden sizes of the last 2 fully connected layers (FC1 and FC2) are 1,024 and 2, respectively. The first row in the figure is with WD *λ* = 0.001, and the second row is with WD *λ* = 0. The network was trained with cosine annealing learning rate scheduler (with initial learning rate *η* = 0.03, ending with *η* = 0.0299).

In Fig. 3, we show the dynamics of *ρ* alongside train loss and test error. We show results with and without WD in the top and bottom rows of Fig. 3, respectively. $L_{S}$ decreases with *μ* increasing and *σ* decreasing. The figures show that in our experiments, the large margins of some of the data points decrease during GD, contributing to a decrease in *σ*. Furthermore, Eq. 11 suggests that for small *ρ*, the term dominating the decrease in $L_{S}$ is −2*ρμ*. For larger *ρ*, the term *ρ*^2^*M* = *ρ*^2^(*σ*^2^ + *μ*^2^) becomes important: Eventually, $L_{S}$ decreases because *σ*^2^ decreases. The regularization term, for standard small values of *λ*, is relevant only in the final phase, when *ρ* is in the order of *ρ*_0_. For *λ* = 0, the loss at the global equilibrium (which happens at *ρ* = *ρ*_0_) is $L_{S}=0$ (since $\mu =\frac{1}{\rho_{0}}$, *M* = *μ*^2^, and *σ*^2^ = 0).

To sum up, starting from small initialization, gradient techniques will explore critical points with *ρ* growing from zero. Thus, quasi-interpolating solutions with small *ρ* (corresponding to large margin solutions) may be found before the many large *ρ* quasi-interpolating solutions that have worse margins (see Fig. 3, top and bottom rows). This dynamics can take place even in the absence of regularization; however, *λ* > 0 makes the process more robust and bias it toward small *ρ*.

## Generalization: Rademacher Complexity of Convolutional Layers

### Classical Rademacher bounds

In this section, we analyze the test performance of the learned neural network. Following the standard learning setting, we assume that there is some underlying distribution *P* of labeled samples (*x*, *y*) and the training data $S={\left\{\left(x_{i},y_{i}\right)\right\}}_{i=1}^{N}$ consist of *N* independent and identically distributed samples from *P*. The model *f_W_* is assumed to perfectly fit the training samples, i.e., *f_W_*(*x_i_*) = *y_i_* = ± 1.

We would like to upper bound the classification error $\mathrm{err}\left(f_{W}\right)≔E_{\left(x,y\right)∼P}\left[I\left[\mathrm{sign}\left(f_{W}\left(x\right)\right)\neq y\right]\right]$ of the learned function *f_W_* in terms of the number of samples *N* and the norm *ρ* of *f_W_*.

This analysis is based on the following data-dependent measure of the complexity of a class of functions.

Definition *Rademacher complexity*. Let ℍ be a set of real-valued functions h:X→ℝ defined over a set X. Given a fixed sample $S\in X^{m}$, the empirical Rademacher complexity of ℍ is defined as follows:

$$R_{S}\left(ℍ\right)≔\frac{2}{m}E_{\sigma }\left[\underset{h\in ℍ}{\sup}|\sum_{i=1}^{m}\sigma_{i}h\left(x_{i}\right)|\right]$$

The expectation is taken over σ = (*σ*_1_, …, *σ_m_*), where, *σ_i_* ∈ {±1} are independent and identically distributed and uniformly distributed samples.

The Rademacher complexity measures the ability of a class of functions to fit noise. The empirical Rademacher complexity has the added advantage that it is data dependent and can be measured from finite samples.

Theorem 3. Let P be a distribution over ℝ*^d^* × {±1}. Let $F=\left\{f_{W}|\prod_{i=1}^{L}∥W_{i}∥\leq 1\right\}$. Let $S={\left\{\left(x_{i},y_{i}\right)\right\}}_{i=1}^{N}$ be a dataset of independent and identically distributed samples selected from P. Then, with probability at least 1 − δ over the selection of S, for any *f_W_ that perfectly fits the data (i.e., f_W_*(*x_i_*) = *y_i_*), we have

$$\mathrm{er}r_{P}\left(f_{W}\right)\leq 2\left(\rho +1\right)\cdot R_{S}\left(F\right)+3\sqrt{\frac{\log\left(2{\left(\rho +1\right)}^{2}/\delta \right)}{2N}}$$ (12)

*Proof.* Let *t* ∈ ℕ ∪ {0} and $G_{t}=\left\{f_{W}|\prod_{i=1}^{L}∥W_{i}{∥}_{2}\in \left[t,t+1\right]\right\}$ We consider the ramp loss function

$$\ell_{\text{ramp}}\left(y,y^{\prime }\right)=\left\{\begin{array}{cc} 1, & \text{if}\;{\mathrm{yy}}^{\prime }\leq 0,\\ 1-{\mathrm{yy}}^{\prime }, & \text{if}\;0\leq {\mathrm{yy}}^{\prime }\leq 1,\\ 0, & \text{if}\;{\mathrm{yy}}^{\prime }\geq 1 \end{array}\right.$$

By Theorem 3.3 in [50], for any *t* ∈ ℕ ∪ {0}, with probability at least $1-\frac{\delta }{t\left(t+1\right)}$, for any function $f_{W}\in G_{t}$, we have

$$E_{\left(x,y\right)}\left[\ell_{\text{ramp}}\left(f_{W}\left(x\right),y\right)\right]\leq \frac{1}{N}\sum_{i=1}^{N}\ell_{\text{ramp}}\left(f_{W}\left(x_{i}\right),y_{i}\right)+2R_{S}\left(G_{t}\right)+3\sqrt{\frac{\log\left(2{\left(t+1\right)}^{2}/\delta \right)}{2N}}$$ (13)

We note that for any function *f_W_* for which *f_W_*(*x_i_*) = *y_i_* = ± 1, we have *ℓ_ramp_*(*f_W_*(*x_i_*), *y_i_*) = 0. In addition, for any function *f_W_* and pair (*x*, *y*), we have *ℓ_ramp_*(*f_W_*(*x*), *y*) ≥ *I*[*sign*(*f_W_*(*x*)) ≠ *y*]. Therefore, we conclude that with probability at least $1-\frac{\delta }{t\left(t+1\right)}$, for any function $f_{W}\in G_{t}$, we have

$$\mathrm{er}r_{P}\left(f_{W}\right)\leq 2R_{S}\left(G_{t}\right)+3\sqrt{\frac{\log\left(2{\left(t+1\right)}^{2}/\delta \right)}{2N}}$$ (14)

We notice that by the homogeneity of ReLU neural networks, we have $R_{S}\left(G_{t}\right)\leq \left(t+1\right)\cdot R_{S}\left(F\right)$. By union bound over all *t* ∈ ℕ ∪ {0}, Eq. 14 holds uniformly for all *t* ∈ ℕ ∪ {0} and $f_{W}\in G_{t}$ with probability at least 1 − *δ*. For each *f_W_* with $\prod_{i=1}^{L}∥W_{i}{∥}_{2}=\rho$, we can apply the bound with *t* = ⌊*ρ*⌋ since $f_{W}\in G_{t}$ and obtain the desired bound,

$$\begin{array}{cc} \mathrm{er}r_{P}\left(f_{W}\right) & \leq 2\left(t+1\right)\cdot R_{S}\left(G_{t}\right)+3\sqrt{\frac{\log\left(2{\left(t+1\right)}^{2}/\delta \right)}{2N}}\\ & \leq 2\left(\rho +1\right)\cdot R_{S}\left(F\right)+3\sqrt{\frac{\log\left(2{\left(\rho +1\right)}^{2}/\delta \right)}{2N}} \end{array}$$ (15)

The above theorem provides an upper bound on the classification error of the trained network *f_W_* that perfectly fits the training samples. The upper bound is decomposed into 2 main terms. The first term is proportional to the norm of the trained model *ρ* and the Rademacher complexity of F that is the set of the normalized neural networks and the second term scales as $\sqrt{\log\left(\rho /\delta \right)/N}$. As shown in Theorem 1 in [51], this term is upper bounded by $R_{S}\left(F\right)\leq \left(\sqrt{2\log\left(2\right)L}+1\right)/\sqrt{\left\{N\right\}}$, assuming that the samples are taken from the *d*-dimensional ball $B_{d}$ of radius 1. The overall bound is then (assuming zero training error)

$$\mathrm{er}r_{P}\left(f_{W}\right)\leq \frac{2\left(\rho +1\right)\left(\sqrt{2\log\left(2\right)L}+1\right)}{\sqrt{N}}+3\sqrt{\frac{\log\left(2{\left(\log\left(\rho \right)+1\right)}^{2}/\delta \right)}{2N}}$$ (16)

We note that while the mentioned bound on ${\mathbb{R}}_{N}\left(F\right)$ depends on the architecture of the network, it does not depend in an explicit way on the training set. However, as shown in Eq. 6 in [51], the bound may be improved further if the matrices’ stable rank is low, which happens with low rank of the weight matrices. In practice, the value of ${\mathbb{R}}_{N}\left(F\right)$ depends not only on the network architecture (e.g., convolutional) but also on the underlying optimization (e.g., *L*_2_ versus *L*_1_) and on the data (e.g., rank).

### Relative generalization

We now consider 2 solutions with zero empirical loss of the square loss regression problem obtained with the same ReLU deep network and corresponding to 2 different minima with 2 different *ρ* values. Let us call them *g^a^*(*x*) = *ρ_a_f^a^*(*x*) and *g^b^*(*x*) = *ρ_b_f^b^*(*x*). Using the notation of this paper, the functions *f_a_* and *f_b_* correspond to networks with normalized weight matrices at each layer.

Let us assume that *ρ_a_* < *ρ_b_*.

We now use Eq. 16 and the fact that the empirical ${\hat{L}}_{\gamma }$ for both functions is the same to write $L_{0}\left(f^{a}\right)=L_{0}\left(F^{a}\right)\leq c_{1}\rho_{a}{\mathbb{R}}_{N}\left(\tilde{F}\right)+c_{2}\sqrt{\frac{\ln\left(\frac{1}{\delta }\right)}{2N}}$ and $L_{0}\left(f^{b}\right)=L_{0}\left(F^{b}\right)\leq c_{1}\rho_{b}{\mathbb{R}}_{N}\left(\tilde{F}\right)+c_{2}\sqrt{\frac{\ln\left(\frac{1}{\delta }\right)}{2N}}$. The bounds have the form

$$L_{0}\left(f^{a}\right)\leq A\rho_{a}+\epsilon ,$$ (17)

and

$$L_{0}\left(f^{b}\right)\leq A\rho_{b}+\epsilon .$$ (18)

Thus, the upper bound for the expected error *L*_0_(*f^a^*) is better than the bound for *L*_0_(*f^b^*). Of course, this is just an upper bound. As a consequence, this result does not guarantee that a solution with smaller *ρ* will always have a smaller expected error than a solution with larger *ρ*.

Notice that this generalization claim is just a relative claim about different solutions obtained with the same network trained on the same training set.

**Fig. 4.**
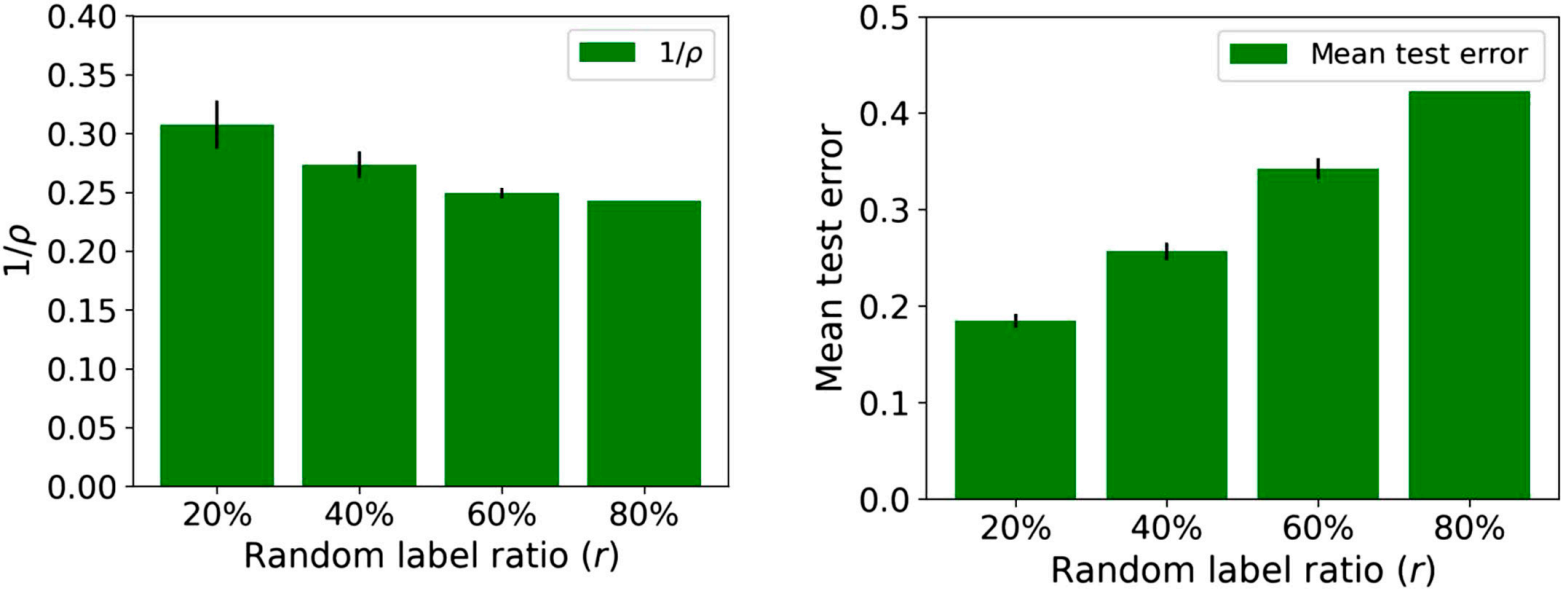
Mean 1/*ρ* and test error results over 10 runs for binary classification on CIFAR10 trained with LM and different percentages of random labels (*r* = 20%, 40%, 60%, and 80%), initialization scale of 1, and WD of 0.001. As mentioned in the text, the norm of the convolutional layers is just the norm of the filters. (Note that this network fails to get convergence with 100% random labels.)

**Fig. 5.**
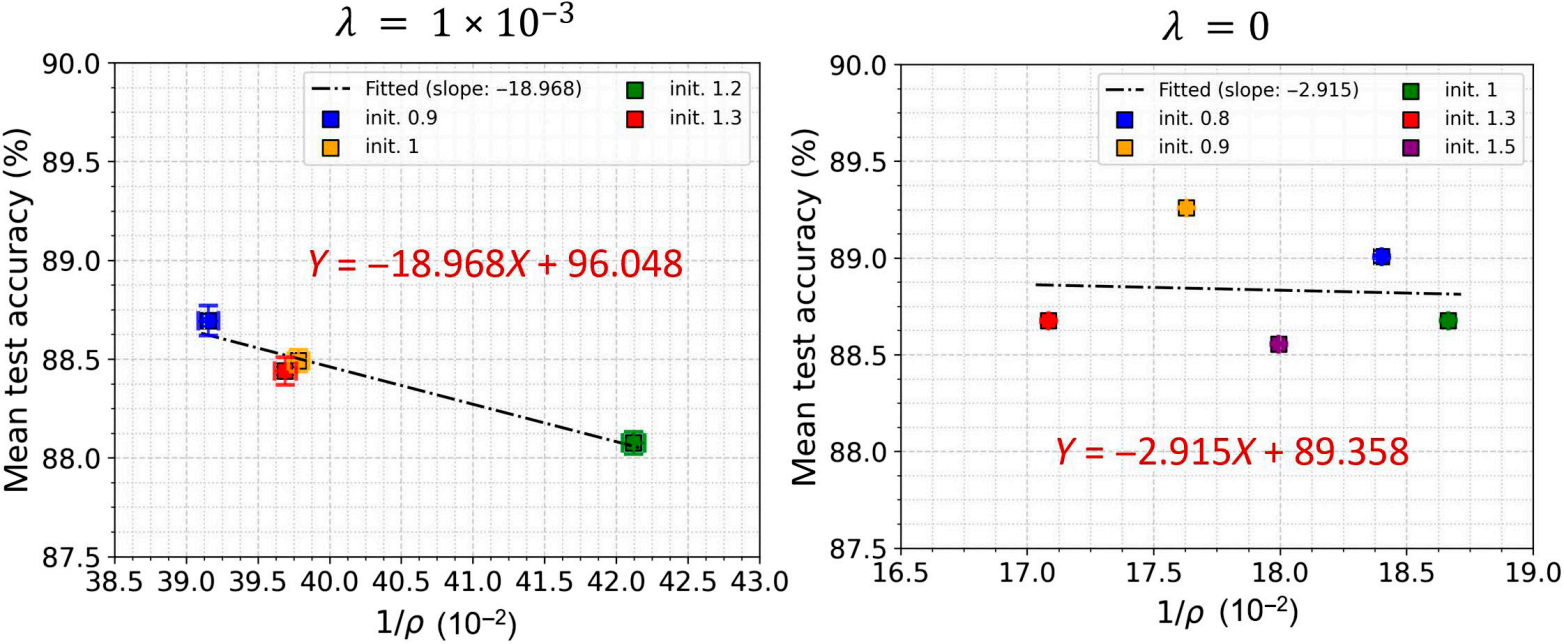
Scatter plots for 1/*ρ* and mean test accuracy based on 10 runs for binary classification on CIFAR10 using LM normalization (LN), square loss, and WD (left) and without WD (right). In the left figure, the network was trained with different initialization scales (init. = [0.9, 1, 1.2, 1.3]) and with WD (λ = 1 × 10^−3^), while in the right figure, the network was trained with init. = [0.8, 0.9, 1, 1.3, 1.5] and no WD (*λ* = 0). The horizontal and vertical error bars correspond to the standard deviations of 1/*ρ* and mean test accuracy computed over 10 runs for different initializations, while the square dots correspond to the mean values. When *λ* > 0, the coefficient (*R*^2^), *P* value and slope for linear regression between 1/*ρ* and mean test accuracy are: *R*^2^ = 0.94, *P* = 0.031, and slope = −18.968; when *λ* = 0, the coefficient *R*^2^ = 0.004, *P* = 0.92, and slope = −2.915.

Figure 4 shows clearly that increasing the percentage of random labels increases the *ρ* that is needed to maintain interpolation—thus decreasing the margin—and that, at the same time, the test error increases, as expected. This monotonic relation between margin and accuracy at test seems to break down for small differences in margin as shown in Fig. 5, although the significance of the effect is unclear. Of course, this kind of behavior is not inconsistent with an upper bound.

### Novel bounds for sparse networks

In the Classical Rademacher bounds section, we describe generic bounds on the Rademacher complexity of deep neural networks. In these cases, *ρ* measures the product of the Frobenius norms of the network’s weight matrices in each layer. For convolutional networks, however, the operation in each layer is computed with a kernel, described by the vector *w*, that acts on each patch of the input separately. Therefore, a convolutional layer is represented by a Toeplitz matrix *W*, whose blocks are each given by *w*. A naive application of Eq. 16 to convolutional networks give a large bound, where the Frobenius norm of the Toeplitz matrix is equivalent to norm of the kernel multiplied by the number of patches.

In this section, we provide an informal analysis of the Rademacher complexity, showing that it can be reduced by exploiting the first one of the 2 properties of convolutional layers: (a) the locality of the convolutional kernels and (b) weight sharing. These properties allow us to bound the Rademacher complexity by taking the products of the norms of the kernel *w* instead of the norm of the associated Toeplitz matrix *W*. Here, we outline the results with more precise statements and proofs to be published separately.

We consider the case of one-dimensional convolutional networks with nonoverlapping patches and one channel per layer. For simplicity, we assume that the input of the network lies in *ℝ^d^*, with *d* = 2*^L^* and the stride and the kernel of each layer are 2. The analysis can be easily extended to kernels of different sizes. This means that the network *h*(*x*) can be represented as a binary tree, where the output neuron is computed as $W^{L}\cdot \sigma \left(v_{1}^{L}\left(x\right),v_{2}^{L}\left(x\right)\right)$, $v_{1}^{L}\left(x\right)=W^{L-1}\cdot \sigma \left(v_{1}^{L-1}\left(x\right),v_{2}^{L-1}\left(x\right)\right)$, $v_{2}^{L}\left(x\right)=W^{L-1}\cdot \sigma \left(v_{3}^{L-1}\left(x\right),v_{4}^{L-1}\left(x\right)\right)$ , and so on. This means that we can write the *i*th row of the Toeplitz matrix of the *l*th layer (0, …, 0, −*W^l^*−, 0…, 0), where *W^l^* appears on the 2*^i^* − 1 and 2*^i^* coordinates. We define a set H of neural networks of this form, where each layer is followed by a ReLU activation function and $\prod_{l=1}^{L}W^{l}\leq \rho$.

Theorem 4. Let H be the set of binary-tree-structured neural networks over *ℝ^d^, with d* = 2*^L^* for some natural number *L*. Let *X* = {*x*_1_, …, *x_N_*} ⊂ *ℝ^d^* be a set of samples. Then,

$$R_{X}\left(H\right)\leq \frac{2^{L}\rho \sqrt{\sum_{i=1}^{N}∥x_{i}{∥}^{2}}}{N}$$ (19)

*Proof sketch.* First, we rewrite the Rademacher complexity in the following manner:

$$\begin{array}{cc} {ℛ}_{X}\left(ℋ\right) & =E_{\epsilon }\underset{h\in ℋ}{\sup}\left|\frac{1}{N}\sum_{i=1}^{N}\epsilon_{i}\cdot h\left(x_{i}\right)\right|\\ & =E_{\epsilon }\underset{h\in ℋ}{\sup}\frac{1}{N}\left|\sum_{i=1}^{N}\epsilon_{i}\cdot W^{L}\cdot \sigma \left(v_{1}\left(x\right),v_{2}\left(x\right)\right)\right|\\ & =E_{\epsilon }\underset{h\in ℋ}{\sup}\frac{1}{N}\sqrt{{\left|\sum_{i=1}^{N}\epsilon_{i}\cdot W^{L}\cdot \sigma \left(v_{1}\left(x\right),v_{2}\left(x\right)\right)\right|}^{2}} \end{array}$$ (20)

Next, by the proof of Lemma 1 in [51], we obtain that

$$\begin{array}{cc} R_{X}\left(H\right) & \leq 2E_{\epsilon }\underset{h\in H}{\sup}\frac{1}{N}\sqrt{∥W^{L}{∥}^{2}\cdot ∥\sum_{i=1}^{N}\epsilon_{i}\left(v_{1}\left(x\right),v_{2}\left(x\right)\right){∥}^{2}}\\ & =E_{\epsilon }\underset{h\in H}{\sup}\frac{1}{N}\sqrt{∥W^{L}{∥}^{2}\cdot \sum_{j=1}^{2}∥\sum_{i=1}^{N}\epsilon_{i}v_{j}\left(x_{i}\right){∥}^{2}} \end{array}$$ (21)

By applying this peeling process *L* times, we obtain the following inequality:

$$\begin{array}{c} R_{X}\left(H\right)\leq 2^{L-1}E_{\epsilon }\underset{h\in H}{\sup}\frac{1}{N}\sqrt{\prod_{l=1}^{L}∥W^{l}{∥}^{2}\cdot \sum_{j=1}^{d}∥\sum_{i=1}^{N}\epsilon_{i}x_{\mathrm{ij}}{∥}^{2}}\\ =2^{L-1}E_{\epsilon }\underset{h\in H}{\sup}\frac{1}{N}\sqrt{\prod_{l=1}^{L}∥W^{l}{∥}^{2}\cdot ∥\sum_{i=1}^{N}\epsilon_{i}x_{i}{∥}^{2}}\\ \leq \frac{2^{L-1}\rho E_{\epsilon }∥\sum_{i=1}^{N}\epsilon_{i}x_{i}∥}{N}\\ \leq \frac{2^{L-1}\rho \sqrt{\sum_{i=1}^{N}∥x_{i}{∥}^{2}}}{N} \end{array}$$ (22)

where the factor 2^*L* − 1^ is obtained because the last layer is linear (see [52]). We note that a better bound can achieved when using the reduction introduced in [51], which would give a factor of $\sqrt{2\text{log}\left(2\right)L}+1$ instead of 2^*L* − 1^.

Thus, one ends up with a bound scaling as the product of the norms of the kernel at each layer. The constants may change depending on the architecture, the number of patches, the size of the patches, and their overlap.

This special nonoverlapping case can be extended to the general convolutional case. In fact, a proof of the following conjecture will be provided in [53].

Conjecture 1. If a convolutional layer has overlap among its patches, then the nonoverlap bound

$$R_{N}\left(H_{L}\right)\leq 2^{L-1}\rho ∥x∥$$ (23)

where *ρ* is the product of the norms of the kernels at each layer becomes

$$R_{N}\left(H_{L}\right)\leq 2^{L-1}\rho \sqrt{\frac{K}{K-O}}∥x∥,$$ (24)

where *K* is the size of the kernel (number of components) and *O* is the size of the overlap.

*Sketch proof.* Call *P* the number of patches and *O* the overlap. With no overlap, then *PK* = *D*, where *D* is the dimensionality of the input to the layer. In general, $P=\frac{D-O}{K-O}$. It follows that a layer with the most overlap can add at most $<∥x∥\sqrt{K}$ to the bound. Notice that we assume that each component of *x_i_* averaged across *i* will have norm $\sqrt{\frac{1}{d}}$.

#### The bound is surprisingly small

In this section, we have derived bounds for convolutional networks that may potentially be orders of magnitude smaller than equivalent similar bounds for dense networks. We note that a naive application of Corollary 2 in [29] for the network that we used in Theorem 4 would require treating the network as if it were a dense network. In this case, the bound would be proportional to the product of the norms of each of the Toeplitz matrices in the network individually. In this case, the total bound becomes

$$\frac{2^{L}\sqrt{\prod_{l=1}^{L}\left(2^{l}\right)}\rho \sqrt{\sum_{i=1}^{N}∥x_{i}{∥}^{2}}}{N}=\frac{2^{0.25L^{2}+1.25L}\rho \sqrt{\sum_{i=1}^{N}∥x_{i}{∥}^{2}}}{N}$$ (25)

which is much larger than the bound we obtained earlier. The key point is that the Rademacher bounds achievable for sparse networks are much smaller than for dense networks. This suggests that convolutional network with local kernels may generalize much better than dense network, which is consistent in spirit with approximation theory results (compositionally sparse target functions can be approximated by sparse networks without incurring in the curse of dimensionality, whereas generic functions cannot be approximated by dense networks without the curse). They also confirm the empirical success of convolutional networks compared to densely connected networks.

**Fig. 6.**
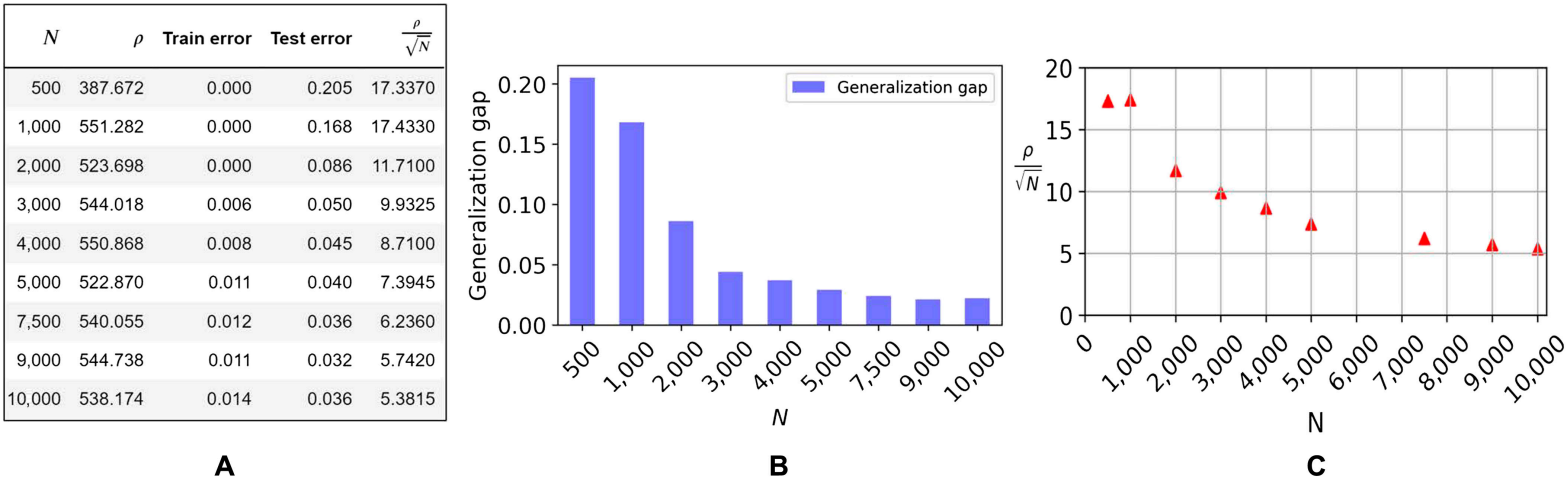
Product norm (*ρ*) and test error with respect to different training data sizes (*N*) for the 6-layer model trained with LM and square loss. The initialization scale is 0.1, WD *λ* = 10^−3^, no biases, the initial learning rate is 0.03 with cosine annealing scheduler; we used the SGD optimizer (momentum =0.9) and test data size =2, 000 in a binary classification task on CIFAR10 dataset. (A) The table shows the product norm *ρ*, mean training errors, mean test errors (average over the last 100 epochs), and generalization upper bound for different *N*. (B) A bar plot for the generalization gap for different *N*. (C) Generalization error upper bound is proportional to ($\frac{\rho }{\sqrt{N}}$). The bounds are vacuous but “only” by an order of magnitude, while other bounds based on the number of parameters (here, 3,519,335) are typically much looser.

**Fig. 7.**
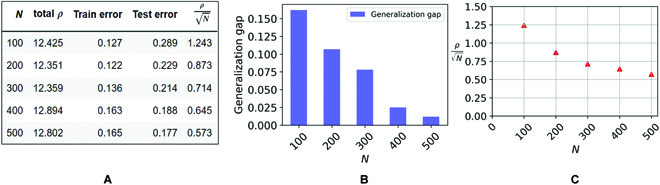
Product norm (*ρ*) and test error with respect to different training data sizes (*N*) for the 3-layer model (with nonoverlapped convolutional image patches, kernel size = 3 × 3, and stride = 3) trained with LM and square loss. The initialization scale is 0.1, WD *λ* = 0.001, no biases, batch size is 32, and the initial learning rate is 0.03 with cosine annealing scheduler; we used the SGD optimizer (momentum = 0.9) and test data size = 2,000 in a binary classification task on CIFAR10 dataset. (A) The table shows the product norm *ρ*, mean training errors, mean test errors (average over the last 100 epochs), and generalization upper bound for different *N* . (B) A bar plot for the generalization gap for different *N* . (C) Generalization error upper bound is a constant (see text) times ($\frac{\rho }{\sqrt{N}}$). The bounds are almost not vacuous depending on the constant (see text).

It is also important to observe that the bounds we obtained may be nonvacuous in the overparameterized case, unlike Vapnik–Chervonenkis bounds that depend on the number of weights and are therefore always vacuous in overparameterized situations. With our norm-based bounds, it is, in principle, possible to have overparametrization and interpolation simultaneously with nonvacuous generalization bounds: This is suggested by Fig. 6. Figure 7 shows the case of a 3-layer convolutional network with a total number of parameters of ≈20,000.

## Neural Collapse

A recent paper [12] described 4 empirical properties of the terminal phase of training (TPT) deep networks, using the cross-entropy loss function. TPT begins at the epoch where training error first vanishes. During TPT, the training error stays effectively zero, while training loss progressively decreases. Direct empirical measurements expose an inductive bias that they call NC, involving 4 interconnected phenomena. Informally, (NC1) cross-example within-class variability of last-layer training activations collapses to zero, as the individual activations themselves collapse to their class means. (NC2) The class means collapse to the vertices of a simplex ETF. (NC3) Up to rescaling, the last-layer classifiers collapse to the class means or, in other words, to the simplex ETF (i.e., to a self-dual configuration). (NC4) For a given activation, the classifier’s decision collapses to simply choose whichever class has the closest train class mean (i.e., the nearest class center decision rule).

We now formally define the 4 NC conditions. We consider a network *f_W_*(*x*) = *W_L_h*(*x*), where *h*(*x*) ∈ *ℝ^p^* denotes the last layer feature embedding of the network and *W_L_* ∈ *ℝ*^*C* × *p*^ contains the parameters of the classifier. The network is trained on a *C*-class classification problem on a balanced dataset $S={\left\{\left(x_{\mathrm{cn}},y_{\mathrm{cn}}\right)\right\}}_{n=1,c=1}^{N,C}$ with *N* samples per class. We can compute the per-class mean of the last layer features as follows:

$$\mu_{c}=\frac{1}{N}\sum_{n=1}^{N}h\left(x_{\mathrm{cn}}\right)$$ (26)

The global mean of all features as follows:

$$\mu_{G}=\frac{1}{C}\sum_{c}\mu_{c}=\frac{1}{\mathrm{NC}}\sum_{c=1,n=1}^{C,N}h\left(x_{\mathrm{cn}}\right).$$

Furthermore, the second-order statistics of the last layer features are computed as follows:

$$\begin{array}{c} \Sigma_{W}=\frac{1}{C}\sum_{c=1}^{C}\frac{1}{N}\sum_{n=1}\left(h\left(x_{\mathrm{cn}}\right)-\mu_{c}\right){\left(h\left(x_{\mathrm{cn}}\right)-\mu_{c}\right)}^{⊤}\\ \Sigma_{B}=\frac{1}{C}\sum_{c=1}^{C}\left(\mu_{c}-\mu_{G}\right){\left(\mu_{c}-\mu_{G}\right)}^{⊤}\\ \Sigma_{T}=\frac{1}{\mathrm{NC}}\sum_{c=1,n=1}^{C,N}\left(h\left(x_{\mathrm{cn}}\right)-\mu_{G}\right){\left(h\left(x_{\mathrm{cn}}\right)-\mu_{G}\right)}^{⊤} \end{array}$$ (27)

Here, Σ*_W_* measures the within-class covariance of the features, Σ*_B_* is the between-class covariance, and Σ*_T_* is the total covariance of the features (Σ*_T_* = Σ*_W_* + Σ*_B_*).

We can now list the formal conditions for NC:

• NC1 (variability collapse). Variability collapse states that the variance of the feature embeddings of samples from the same class tends to zero, or formally, *Tr*(Σ*_W_*) → 0.
• NC2 (convergence to simplex ETF). |∥*μ_c_* − *μ_G_*∥_2_ − ∥ *μ*_*c*′_ − *μ_G_*∥_2_| → 0, or the centered class means of the last layer features become equinorm. Moreover, if we define ${\tilde{\mu }}_{c}=\frac{\mu_{c}-\mu_{G}}{∥\mu_{c}-\mu_{G}{∥}_{2}}$, then we have $\langle {\tilde{\mu }}_{c},{\tilde{\mu }}_{c\prime }\rangle =-\frac{1}{C-1}$ for *c* ≠ *c*^′^, or the centered class means are also equiangular. The equinorm condition also implies that $\sum_{c}{\tilde{\mu }}_{c}=0$, i.e., the centered features lie on a simplex.
• NC3 (self-duality). If we collect the centered class means into a matrix *M* = [*μ_c_* − *μ_G_*], then we have $\left|\left|\frac{W^{⊤}}{∥W{∥}_{F}}-\frac{M}{∥M{∥}_{F}}\right|\right|\to 0$, or the classifier *W* and the last layer feature means *M* become duals of each other.
• NC4 (nearest center classification). The classifier implemented by the deep network eventually boils down to choosing the closest mean last layer feature $\text{argma}x_{c}\langle W_{L}^{c},h\left(x\right)\rangle \to \text{argmi}n_{c}∥h\left(x\right)-\mu_{c}{∥}_{2}$.

### Related Work on NC

Since the empirical observation of NC was made in [12], a number of papers have studied the phenomenon in the so-called unconstrained features regime [32–34,39,40]. The basic assumption underlying these proofs is that the features of a deep network at the last layer can essentially be treated as free optimization variables, which converts the problem of finding the parameters of a deep network that minimize the training loss, into a matrix factorization problem of factoring one-hot class labels *Y* ∈ *ℝ*^*C* × *CN*^ into the last layer weights *W* ∈ *ℝ*^*C* × *p*^ and the last layer features *H* ∈ *ℝ*^*p* × *CN*^. In the case of the squared loss, the problem that they study is *min*_*W*, *H*_ ∥ *WH* − *Y*∥^2^ + *λ_W_* ∥ *W*∥^2^ + *λ_H_* ∥ *H*∥^2^.

In this section, we show instead that we can predict the existence of NC and its properties as a consequence of our analysis of the dynamics of SGD on deep binary classifiers trained on the square loss function with LN and WD without any additional assumption. We first consider the case of binary classification and show that NC follows from the analysis of the dynamics of the square loss in the previous sections. The loss function is the same one defined in Eq. 1, and we consider minimization using SGD with a batch size of 1. After establishing NC in this familiar setting, we consider the multiclass setting where we derive the conditions of NC from an analysis of the squared loss function with WD and WN.

### Binary classification

We prove in this section that NC follows from the following property of the landscape of the squared loss that we analyzed in the previous section:

Property 1 [symmetric quasi-interpolation (binary classification)]. Consider a binary classification problem with inputs in a feature space X and label space {+1, −1}. A classifier $f_{W}:X\to \mathbb{R}$ symmetrically quasi-interpolates a training dataset $S={\left\{\left(x_{n},y_{n}\right)\right\}}_{n=1}^{N}$ if, for all training examples, ${{\bar{f}}_{W}}_{n}=y_{n}f_{W}\left(x_{n}\right)=1-\epsilon$, where ϵ is the interpolation gap.

We prove first that the property follows without any assumption at convergence from our previous analysis of the landscape of the squared loss for binary classification.

Lemma 6. An overparameterized deep ReLU network for binary classification trained to convergence under the squared loss in the presence of WD and WN satisfies the symmetric quasi-interpolation property. Furthermore, the gap to interpolation of the regularized network is $\epsilon =\frac{\lambda }{\mu^{2}+\lambda }$ *where* $\mu =\frac{1}{N}\sum_{i}{\bar{f}}_{i}$*.*

*Proof.* Consider the regularized square loss $L_{S}=\frac{1}{N}\sum_{i=1}^{N}{\left(\rho {\bar{f}}_{i}-1\right)}^{2}+\lambda \rho^{2}$. We recall the definitions made earlier in in the Classification with square loss minimization" section of the margin ${\bar{f}}_{i}=y_{i}f_{i}$, and the first- and second-order sample statistics of the margin $\mu =\frac{1}{N}\sum_{i=1}^{N}{\bar{f}}_{i},M=\frac{1}{N}\sum_{i=1}^{N}{\bar{f}}_{i}^{2},\sigma^{2}=M-\mu^{2}$. We consider deep networks that are sufficiently overparameterized to attain 100% accuracy on the training dataset, which means ${\bar{f}}_{i}>0$. Since the weights of the deep network ${\left\{V_{k}\right\}}_{k=1}^{L}$ are normalized and the data *x_i_* lie within the unit norm ball, we have that $|{\bar{f}}_{i}|\leq 1$. Although ${\bar{f}}_{i}$ could take values close to 1, the typically observed values of ${\bar{f}}_{i}$ in our experiments are approximately 5 × 10^−3^. For our purposes, it suffices to note that there exists a maximum possible margin, such that $0<{\bar{f}}_{i}\leq \bar{\mu }$ for all training examples for a given dataset and network architecture.

Using these definitions, we can rewrite the deep network training problem as follows:

$${\text{min}}_{\rho ,{\left\{V_{k}\right\}}_{k=1}^{L}}L_{S}=\left(M+\lambda \right)\rho^{2}-2\rho \mu +1.$$ (28)

All critical points (including minima) need to satisfy $\frac{\partial L_{S}}{\partial \rho }=0$, from which we get $\rho =\frac{\mu }{M+\lambda }$. If we plug this back into the loss, then our minimization problem becomes:

$$\begin{array}{c} {\text{min}}_{{\left\{V_{k}\right\}}_{k=1}^{L}}\left(M+\lambda \right){\left(\frac{\mu }{M+\lambda }\right)}^{2}-2\frac{\mu^{2}}{M+\lambda }+1\\ ={\text{min}}_{{\left\{V_{k}\right\}}_{k=1}^{L}}1-\frac{\mu^{2}}{M+\lambda }\\ ={\text{min}}_{{\left\{V_{k}\right\}}_{k=1}^{L}}\frac{\sigma^{2}+\lambda }{\mu^{2}+\sigma^{2}+\lambda }\\ ={\text{min}}_{{\left\{V_{k}\right\}}_{k=1}^{L}}\frac{1}{1+\frac{\mu^{2}}{\sigma^{2}+\lambda }} \end{array}$$ (29)

Hence, to minimize the loss, we have to find ${\left\{V_{k}\right\}}_{k=1}^{L}$ that maximizes *μ*^2^ and minimizes *σ*^2^. Since we assumed that the network is expressive enough to attain any value, the loss is minimized when *σ*^2^ = 0 and $\mu =\bar{\mu }$. Thus, all training examples have the same margin.

If *σ*^2^ → 0, then all margins tend to the same value, ${\bar{f}}_{i}\to \bar{\mu }$, and the optimum value of *ρ* is $\rho =\frac{\bar{\mu }}{{\bar{\mu }}^{2}+\lambda }$. This means that the gap to interpolation is $\epsilon =1-\rho \bar{\mu }=\frac{\lambda }{\lambda +{\bar{\mu }}^{2}}$.

**Fig. 8.**
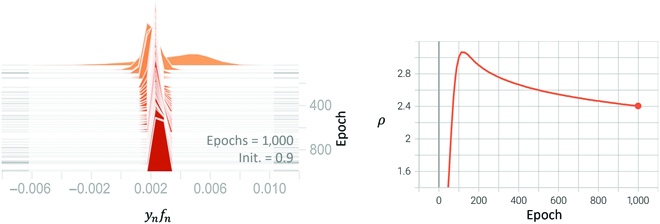
Histogram of *y_n_f_n_* across 1,000 training epochs for binary classification on the CIFAR10 dataset with LM and WD (*λ*) = 0.001, initial learning rate of 0.03, and initialization of 0.9. The histogram narrows as training progresses. The final histogram (in red) is concentrated, as expected for the emergence of NC1. The right side of the plot shows the time course of the top *ρ* over the same 1,000 epochs.

The prediction *σ* → 0 has empirical support: we show in Fig. 8 that all the margins converge to be roughly equal. Once within-class variability disappears and for all training samples, the last layer features collapse to their mean. The outputs and margins then also collapse to the same value. We can see this in the left plot of Fig. 10 where all of the margin histograms are concentrated around a single value. We visualize the evolution of the training margins over the training epochs in Fig. 8, which shows that the margin distribution concentrates over time. At the final epoch, the margin distribution (colored in yellow) is much narrower than at any intermediate epochs. Notice that our analysis of the origin of the SGD noise shows that “strict” NC1 never happens with SGD, in the sense that the margins are never, not even asymptotically, exactly equal to each other but just very close.

We now prove that NC follows from Property 1.

Theorem 5. Let $S={\left\{\left(x_{n},y_{n}\right)\right\}}_{n=1}^{N}$ be a dataset. Let (*ρ*, *V*) be the parameters of a ReLU network f, such that *V_L_* has converged when training using SGD with batches of size 1 on the square loss with LN + WD. Let $\mu_{+}=\frac{1}{N}\sum_{n=1,y_{n}=1}^{N}h\left(x_{n}\right)$*,* $\mu_{-}=\frac{1}{N}\sum_{n=1,y_{n}=-1}^{N}h\left(x_{n}\right)$. Consider points of convergence of SGD that satisfy *Property 1*. Those points also satisfy the conditions of NC as described below.

• *NC1: μ*_+_ = *h*(*x_n_*) for all n ∈ [*N*], *y_n_* = 1*, μ*_−_ = *h*(*x_n_*) for all n ∈ [*N*], *y_n_* = −1*.*

• *NC2: μ*_+_ = −*μ*_−_, which is the structure of an ETF with 2 vectors.

• *NC3: V_L_* ∝ *μ*_+_, *μ*_−_*.*

• *NC4: sign*(*ρf_V_*(*x*)) = *arg min*_*c* ∈ {+1, −1}_ ∥ *μ_c_* − *h*(*x*)∥*.*

*Proof*. The update equations for SGD on the square loss function with LN+WD are given by:

$$\begin{array}{c} V_{L}^{\left(t+1\right)}=V_{L}^{\left(t\right)}-\eta \frac{\partial L}{\partial V_{L}^{\left(t\right)}}\\ ⟹V_{L}^{\left(t+1\right)}=V_{L}^{\left(t\right)}-\eta \times \left(2\rho \left(\rho {\bar{f}}_{n}-1\right)y_{n}h\left(x_{n}\right)+2\nu_{L}^{\left(t\right)}V_{L}^{\left(t\right)}\right) \end{array}$$ (30)

We can apply the unit norm constraints ${\left\|V_{L}^{\left(t+1\right)}\right\|}^{2}=1$ and ${\left\|V_{L}^{\left(t\right)}\right\|}^{2}=1$ and ignore all terms that are *O*(*η*^2^) to compute $\nu_{L}^{\left(t\right)}$ as:

$$\begin{array}{c} 2\nu_{L}^{\left(t\right)}=2\rho y_{n}V_{L}^{\left(t\right)⊤}h\left(x_{n}\right)\left(1-\rho {\bar{f}}_{n}\right)\\ ⟹\nu_{L}^{\left(t\right)}=\rho {\bar{f}}_{n}\left(1-\rho {\bar{f}}_{n}\right) \end{array}.$$ (31)

This gives us the following SGD update:

$$V_{L}^{\left(t+1\right)}=V_{L}^{\left(t\right)}-\eta \times 2\rho y_{n}\left(\rho {\bar{f}}_{n}-1\right)\left(h\left(x_{n}\right)-f_{n}V_{L}^{\left(t\right)}\right).$$ (32)

Using Property 1, we can see that for every training sample in class *y_n_* = 1, $h\left(x_{n}\right)=\frac{\left(1-\epsilon \right)}{\rho }V_{L}$ and for every training sample in class *y_n_* = −1, $h\left(x_{n}\right)=\frac{\left(-1+\epsilon \right)}{\rho }V_{L}$. This shows that within-class variability has collapsed and that all last layer features collapse to their mean, which is the condition for NC1. We can also see that *μ*_+_ = −*μ*_−_, which is the condition for NC2 when there are 2 vectors in the simplex ETF. The SGD convergence condition also tells us that *V_L_* ∝ *μ*_+_ and *V_L_* ∝ *μ*_−_, which gives us the NC3 condition. NC4 follows then from NC1 to NC2, as shown by theorems in [12].

### Multiclass classification

**Fig. 9.**
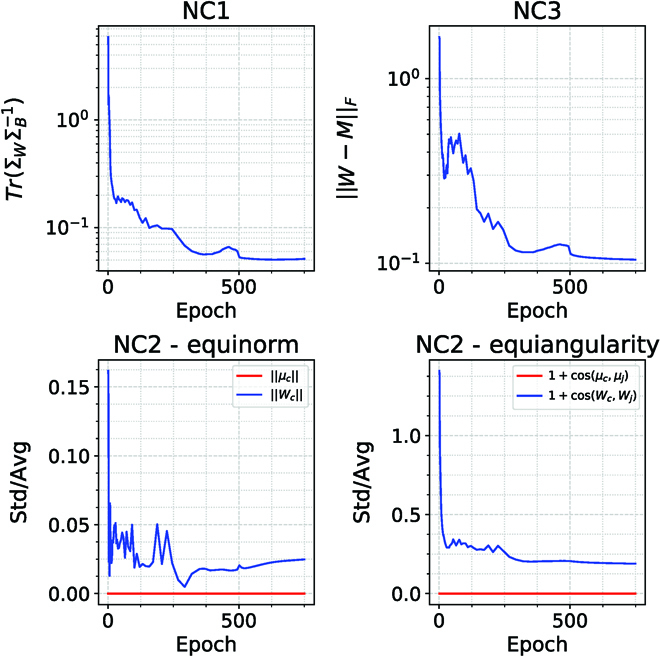
NC occurs during training for binary classification. This figure is similar to other published results on NC, such as for instance [12] for the case of exponential-type loss function. The key conditions for NC are: (a) NC1—variability collapse, which is measured by $\mathrm{Tr}\left(\Sigma_{W}\Sigma_{B}^{-1}\right)$, where Σ*_W_* and Σ*_B_* are the within and between class covariances, (b) NC2—equinorm and equiangularity of the mean features {*μ_c_*} and classifiers {*W_c_*}. We measure the equinorm condition by the standard deviation of the norms of the means (in red) and classifiers (in blue) across classes, divided by the average of the norms, and the equiangularity condition by the standard deviation of the inner products of the normalized means (in red) and the normalized classifiers (in blue), divided by the average inner product (this figure is similar to Fig. 4 in [12]; notice the small scale of the fluctuations), and (c) NC3—self-duality or the distance between the normalized classifiers and mean features. This network was trained on 2 classes of CIFAR10 with WN and WD = 5 × 10^−4^ and learning rate of 0.067, for 750 epochs with a stepped learning rate decay schedule.

In the previous section, we proved the emergence of NC in the case of a binary classifier with scalar outputs, to be consistent with our framework in Problem Setup. The phenomenon of NC was, however, defined in [12] for the case of multiclass classification with deep networks. In this section, we describe how NC emerges in this setting from the minimization of the squared loss with WN and WD regularization. We also show in Fig. 9 that our networks show NC, similar to experiments reported in [12].

We consider a classification problem with *C* classes with a balanced training dataset $S=\cup_{c=1}^{C}S_{c}=\cup_{c=1}^{C}{\left\{\left(x_{\mathrm{cn}},c\right)\right\}}_{n=1}^{N}=\left\{\left(x_{n},y_{n}\right)\right\}$ that has *N* training examples $S_{c}={\left\{\left(x_{\mathrm{cn}},c\right)\right\}}_{n=1}^{N}$ per-class *c* ∈ [*C*]. The labels are represented by the unit vectors ${\left\{e_{c}\right\}}_{c=1}^{C}$ in *ℝ^C^*. Since we consider deep homogeneous networks that do not have bias vectors, we center the one-hot labels and scale them so that they have maximum output 1. We denote the resulting labels (for a class-balanced dataset) as ${\tilde{e}}_{c}=\left[\frac{-1}{C-1,}\ldots ,\frac{-1}{C-1},1,\frac{-1}{C-1},\ldots ,\frac{-1}{C-1}\right]$, where the *c*th coordinate is 1. We consider a deep ReLU network *f_W_* : *ℝ^d^* → *ℝ^C^*, which takes the form of *f_W_*(*x*) = *W_L_σ*(*W*_*L* − 1_…*W*_2_*σ*(*W*_1_*x*)…). However, we stick to the normalized reparameterization of the deep ReLU network as *f*(*x*) = *ρV_L_σ*(*V*_*L* − 1_…*V*_2_*σ*(*V*_1_*x*)…). We train this normalized network with SGD on the square loss with LMs and WD. This architecture differs from the one considered the Gradient dynamics section in that it has *C* outputs instead of a scalar output. Let the output of the network be $\rho f_{V}\left(x\right)={\left[\rho f_{V}^{\left(1\right)}\left(x\right)\ldots \rho f_{V}^{\left(C\right)}\left(x\right)\right]}^{⊤}$ and the target vectors be $y_{n}={\left[y_{n}^{\left(1\right)}\ldots y_{n}^{\left(C\right)}\right]}^{⊤}$. We will also follow the notation of [12] and use *h* : *ℝ^d^* → *ℝ^p^* to denote the last layer features of the deep network. This means that $f_{V}^{\left(c\right)}\left(x\right)=\langle V_{L}^{c},h\left(x\right)\rangle$. The squared loss function with WD is written as $L_{S}\left(\rho ,{\left\{V_{k}\right\}}_{k=1}^{L}\right)=\frac{1}{\mathrm{NC}}\sum_{c=1}^{C}\sum_{n=1}^{N}{\left\|y_{\mathrm{cn}}-\rho f_{V}\left(x_{\mathrm{cn}}\right)\right\|}^{2}+\lambda \rho^{2}$.

Property 2 [symmetric quasi-interpolation (multiclass classification)]. Consider a C-class classification problem with inputs in a feature space X and label space ℝ*^C^*. A classifier $f:X\to {\mathbb{R}}^{C}$ symmetrically quasi-interpolates a training dataset $S=\cup_{c=1}^{C}S_{c}=\cup_{c=1}^{C}{\left\{\left(x_{\mathrm{cn}},y_{\mathrm{cn}}\right)\right\}}_{n=1}^{N}$ if, for all training examples, *x_cn_,* $f\left(x_{\mathrm{cn}}\right)\propto {\tilde{e}}_{c}$*.*

Similar to the binary classification case, we show that this property arises from an analysis of the squared loss landscape for multiclass classification.

Lemma 7. An overparameterized deep ReLU classifier trained to convergence under the squared loss in the presence of WD and WN satisfies the symmetric quasi-interpolation property

*Proof*. Consider the regularized square loss $L_{S}=\frac{1}{\mathrm{CN}}\sum_{c=1}^{C}\sum_{n=1}^{N}∥\rho f_{V}\left(x_{\mathrm{cn}}\right)-{\tilde{e}}_{c}{∥}^{2}+\lambda \rho^{2}$. In the multiclass case, we define the first-order statistics of the output of the normalized network as $\mu =\frac{1}{\mathrm{CN}}\sum_{c=1}^{C}\sum_{n=1}^{N}\langle f_{V}\left(x_{\mathrm{cn}}\right),{\tilde{e}}_{c}\rangle$ and $M=\frac{1}{\mathrm{CN}}\sum_{c=1}^{C}\sum_{n=1}^{N}∥f_{V}\left(x_{\mathrm{cn}}\right){∥}^{2}$. We consider deep networks that are overparameterized enough to attain 100% accuracy on the training dataset, which means $\langle f_{V}\left(x_{\mathrm{cn}}\right),{\tilde{e}}_{c}\rangle >0$. Since the weights of the deep network ${\left\{V_{k}\right\}}_{k=1}^{L}$ are normalized and the data *x_cn_* lie within the unit norm ball, we also have that ∥*f_V_*(*x_cn_*) ∥ ≤ 1. However, similar to the binary case, we observe that the norm of *f_V_*(*x_cn_*) takes values of the order of 10^−3^.

Using these definitions, we can rewrite the deep network training problem as:

$${\text{min}}_{\rho ,{\left\{V_{k}\right\}}_{k=1}^{L}}L_{S}=\left(M+\lambda \right)\rho^{2}-2\rho \mu +\frac{C}{C-1}$$ (33)

All critical points (including minima) need to satisfy $\frac{\partial L_{S}}{\partial \rho }=0$, from which we get $\rho =\frac{\mu }{M+\lambda }$. If we plug this back into the loss, then our minimization problem becomes:

$$\begin{array}{c} {\text{min}}_{{\left\{V_{k}\right\}}_{k=1}^{L}}\left(M+\lambda \right)\times {\left(\frac{\mu }{M+\lambda }\right)}^{2}-2\frac{\mu^{2}}{M+\lambda }+\frac{C}{C-1}\\ ={\text{min}}_{{\left\{V_{k}\right\}}_{k=1}^{L}}\frac{C}{C-1}-\frac{\mu^{2}}{M+\lambda } \end{array}$$ (34)

Hence, to minimize the loss we have to find ${\left\{V_{k}\right\}}_{k=1}^{L}$ that maximizes $\frac{\mu^{2}}{M+\lambda }$. Since the network is expressive enough to attain any value and the norm of *f_V_*(*x_cn_*) is bounded, we see that the loss is minimized when *μ*^2^ is maximized. That is, when $f\left(x_{\mathrm{cn}}\right)\propto {\tilde{e}}_{c}$ for all training examples.

We now consider the optimization of the squared loss on deep networks with WN and WD:

$$L_{S}\left(\rho ,{\left\{V_{k}\right\}}_{k=1}^{L}\right)=\frac{1}{\mathrm{NC}}\sum_{c=1}^{C}\sum_{n=1}^{N}{\left\|y_{\mathrm{cn}}-\rho f_{V}\left(x_{\mathrm{cn}}\right)\right\|}^{2}+\sum_{k=1}^{L}\nu_{k}\left({\left\|V_{k}\right\|}^{2}-1\right)+\lambda \rho^{2}$$ (35)

At each time point *t*, the optimization process selects a random class-balanced batch $S^{\prime }=\cup_{c=1}^{C}\cup_{n=1}^{b}S_{c}^{\prime }$ including *B* samples per-class from $S_{c}^{\prime }\subset S_{c}$ and updates the scale and weights of the network is the following manner $V\leftarrow V-\eta \frac{\partial L_{S\prime }\left(\rho ,V\right)}{\partial V}$ and $\rho \leftarrow \rho -\eta \frac{\partial L_{S\prime }\left(\rho ,V\right)}{\partial \rho }$, where *η* > 0 is a predefined learning rate and *b* is a divisor of *N*. A convergence point of the optimization process is a point (*ρ*, *V*) that will never be updated by any possible sequence of steps taken by the optimization algorithm. Specifically, the convergence points of the proposed method are all points *ρ*, *V* for which $\frac{\partial L_{S\prime }\left(\rho ,V\right)}{\partial V}=0$ and $\frac{\partial L_{S\prime }\left(\rho ,V\right)}{\partial \rho }=0$ for all class-balanced batches $S^{\prime }\subset S$.

Theorem 6. Let $S=\cup_{c=1}^{C}{\left\{\left(x_{\mathrm{cn}},c\right)\right\}}_{n=1}^{N}$ be a dataset and B be a divisor of N. Let (*ρ*, *V*) be the parameters of a ReLU network *f_W_, such that V_L_ has converged when training using SGD with balanced batches of size B* = bC on the square loss with LN + WD. Let $\mu_{c}=\frac{1}{N}\sum_{n=1}^{N}h\left(x_{\mathrm{cn}}\right)$*,* $\mu_{G}=\frac{1}{C}\sum_{c=1}^{C}\mu_{c}$*, and M* = […*μ_c_* − *μ_G_*…] ∈ *ℝ*^*p* × *C*^. Consider points of convergence of SGD that satisfy *Property 2*. Then, those points also satisfy the conditions of NC as described below.

• *NC1: μ_c_* = *h*(*x_cn_*) *for all n* ∈ [*N*]*.*

*• NC2: The vectors* ${\left\{\mu_{c}-\mu_{G}\right\}}_{c=1}^{C}$ *form an ETF.*

*• NC3:* $V_{L}^{⊤}=\frac{M}{∥M{∥}_{F}}$*.*

*• NC4:* $\arg\;\max_{c\in \left[C\right]}f_{W}^{c}\left(x\right)=\arg\;\min_{c\in \left[C\right]}∥\mu_{c}-h\left(x\right)∥$*.*

*Proof*. Our training objective is the loss function described in Eq. 35. The network is trained using SGD along with LN and WD. We use SGD with balanced batches to train the network. Each step taken by SGD takes the form $-\eta \frac{\partial L_{S^{\prime }}}{\partial V}$, where $S^{\prime }\subset S$ is a balanced batch containing exactly *b* samples per class. We consider limit points of the learning procedure, meaning that $\frac{\partial L_{S^{\prime }}}{\partial V}=0$ for all balanced batches $S^{\prime }$. Let $S^{\prime }=\cup_{c=1}^{C}\cup_{n=1}^{b}\left\{\left({\hat{x}}_{\mathrm{cn}},{\hat{y}}_{\mathrm{cn}}\right)\right\}$ be such a balanced batch. We use SGD, where, at each time *t*, the batch $S^{\prime }$ is drawn at random from S, to study the time evolution of the normalized parameters *V_L_* in the limit *η* → 0.

$$\begin{array}{c} V_{L}^{\left(t+1\right)}=V_{L}^{\left(t\right)}-\eta \frac{\partial L_{S\prime }}{\partial V_{L}^{\left(t\right)}}\\ V_{L}^{\left(t+1\right)}=V_{L}^{\left(t\right)}-\eta \times \left(\frac{1}{B}\sum_{c\prime =1}^{C}\sum_{n=1}^{b}2\rho \left(\rho f_{V}\left(x_{c\prime n}\right)-{\tilde{e}}_{c\prime }\right)h{\left(x_{c\prime n}\right)}^{⊤}+2\nu_{L}^{\left(t\right)}V_{L}^{\left(t\right)}\right) \end{array}$$ (36)

We can apply the unit norm constraints ${\left\|V_{L}^{\left(t+1\right)}\right\|}_{F}^{2}=$ and and ignore all terms that are *O*(*η*^2^) to compute $\nu_{L}^{\left(t\right)}$ as:

$$\begin{array}{cc} 2\nu_{L}^{\left(t\right)} & =-\frac{1}{B}\sum_{c\prime =1}^{C}\sum_{n=1}^{b}2\rho \mathrm{tr}\left(V_{L}^{\left(t\right)⊤}\left(\rho f_{V}\left(x_{c\prime n}\right)-{\tilde{e}}_{c\prime }\right)h{\left(x_{c\prime n}\right)}^{⊤}\right)\\ ⟹\nu_{L}^{\left(t\right)} & =-\frac{1}{B}\sum_{c\prime =1}^{C}\sum_{n=1}^{b}\rho \mathrm{tr}\left({\left(V_{L}^{\left(t\right)}h\left(x_{c\prime n}\right)\right)}^{⊤}\left(\rho f_{V}\left(x_{c\prime n}\right)-{\tilde{e}}_{c\prime }\right)\right)\\ & =-\frac{1}{B}\sum_{c\prime =1}^{C}\sum_{n=1}^{b}\rho f_{V}{\left(x_{c\prime n}\right)}^{⊤}\left(\rho f_{V}\left(x_{c\prime n}\right)-{\tilde{e}}_{c\prime }\right) \end{array}$$ (37)

This means that the (stochastic) gradient of the loss with respect to the last layer *V_L_* and each classifier vector $V_{L}^{c}$ with LN can be written as (we drop the time index *t* for clarity):

$$\begin{array}{c} \frac{\partial L_{S\prime }}{\partial V_{L}}=\frac{-2\rho }{B}\sum_{c\prime =1}^{C}\sum_{n=1}^{b}f_{V}{\left(x_{c\prime n}\right)}^{⊤}\left(\rho f_{V}\left(x_{c\prime n}\right)-{\tilde{e}}_{c\prime }\right)V_{L}-\left(\rho f_{V}\left(x_{c\prime n}\right)-{\tilde{e}}_{c\prime }\right)h{\left(x_{c\prime n}\right)}^{⊤}\\ \frac{\partial L_{S\prime }}{\partial V_{L}^{c}}=\frac{-2\rho }{B}\sum_{c\prime =1}^{C}\sum_{n=1}^{b}f_{V}{\left(x_{c\prime n}\right)}^{⊤}\left(\rho f_{V}\left(x_{c\prime n}\right)-{\tilde{e}}_{c\prime }\right)V_{L}^{c}-\left(\rho f_{V}^{\left(c\right)}\left(x_{c\prime n}\right)-{\tilde{e}}_{c\prime }^{\left(c\right)}\right)h\left(x_{c\prime n}\right) \end{array}$$ (38)

Let us analyze the equilibrium parameters at the last layer, considering each classifier vector $V_{L}^{c}$ of *V_L_*, separately:

$$\begin{array}{c} 0=\frac{\partial L_{S\prime }}{\partial V_{L}^{c}}=\frac{-2\rho }{B}\sum_{c\prime =1}^{C}\sum_{n=1}^{b}f_{V}{\left(x_{c\prime n}\right)}^{⊤}\left(\rho f_{V}\left(x_{c\prime n}\right)-{\tilde{e}}_{c\prime }\right)V_{L}^{c}-\left(\rho f_{V}^{\left(c\right)}\left(x_{c\prime n}\right)-{\tilde{e}}_{c\prime }^{\left(c\right)}\right)h\left(x_{c\prime n}\right)\\ =\frac{-2\rho }{B}\sum_{n=1}^{b}f_{V}{\left(x_{\mathrm{cn}}\right)}^{⊤}\left(\rho f_{V}\left(x_{\mathrm{cn}}\right)-{\tilde{e}}_{c}\right)V_{L}^{c}-\left(\rho f_{V}^{\left(c\right)}\left(x_{\mathrm{cn}}\right)-1\right)h\left(x_{\mathrm{cn}}\right)\\ -\frac{2\rho }{B}\sum_{c\prime \in \left[C\right]\backslash \left\{c\right\}}\sum_{n=1}^{b}f_{V}{\left(x_{c\prime n}\right)}^{⊤}\left(\rho f_{V}\left(x_{c\prime n}\right)-{\tilde{e}}_{c\prime }\right)V_{L}^{c}-\left(\rho f_{V}^{\left(c\right)}\left(x_{c\prime n}\right)+\frac{1}{C-1}\right)h\left(x_{c\prime n}\right) \end{array}$$ (39)

Using Property 2 and considering solutions that achieve symmetric quasi-interpolation, with $\rho f_{V}\left({\hat{x}}_{\mathrm{cn}}\right)=\alpha {\tilde{e}}_{c}$, we have

$$\frac{2\rho }{B}\sum_{n=1}^{b}\left(\alpha -1\right)h\left(x_{\mathrm{cn}}\right)-\frac{2\rho }{B}\sum_{c\prime \in \left[C\right]\backslash \left\{c\right\}}\sum_{n=1}^{b}\frac{\alpha -1}{C-1}h\left(x_{c\prime n}\right)-\frac{2\alpha \left(\alpha -1\right)C}{C-1}V_{L}^{c}=0$$ (40)

In addition, consider a second batch $S^{\prime \prime }$ that differs from $S^{\prime }$ by only one sample *x*′*_cn_* instead of *x_cn_* from class *c*. By applying the previous Eq. 40 for $S^{\prime }$ and $S^{\prime \prime }$, we can obtain *h*(*x_cn_*) = *h*(*x*′*_cn_*), which proves NC1.

Let $S=\cup_{i=1}^{k}S^{i}$ be a partition of S into *k* = *N*/*b* (an integer) disjoint batches. Since our data are balanced, we obtain that

$$\begin{array}{cc} 0 & =\frac{1}{k}\sum_{i=1}^{k}\frac{\partial L_{S^{i}}\left(\rho ,V\right)}{\partial V_{L}^{c}}\\ & =\frac{\partial L_{S}\left(\rho ,\;V\right)}{\partial V_{L}^{c}}\\ & =\frac{2\rho }{\mathrm{NC}}\sum_{c^{\prime }=1}^{C}\sum_{n=1}^{N}f_{V}{\left(x_{c^{\prime }n}\right)}^{⊤}\left(\rho f_{V}\left(x_{c^{\prime }n}\right)-{\tilde{e}}_{c^{\prime }}\right)V_{L}^{c}-\left(\rho f_{V}^{\left(c\right)}\left(x_{c^{\prime }n}\right)-{\tilde{e}}_{c^{\prime }}^{\left(c\right)}\right)h\left(x_{c^{\prime }n}\right)\\ & =\frac{2\rho }{\mathrm{NC}}\sum_{n=1}^{N}\left(\alpha -1\right)h\left(x_{\mathrm{cn}}\right)-\frac{2\rho }{\mathrm{NC}}\sum_{c^{\prime }\in \left[C\right]\backslash \left\{c\right\}}\sum_{n=1}^{N}\frac{\alpha -1}{C-1}h\left(x_{c^{\prime }n}\right)-\frac{2\alpha \left(\alpha -1\right)C}{C-1}V_{L}^{c} \end{array}$$ (41)

Under the conditions of NC1, we can simply write *μ_c_* = *h*(*x_cn_*) for all *n* ∈ [*N*] and *c* ∈ [*C*]. Let us denote the global feature mean by $\mu_{G}=\frac{1}{C}\sum_{c=1}^{C}\mu_{c}$. This means we have:

$$\frac{\partial L_{S}(\rho V)}{\partial V_{L}^{c}}=0⟹V_{L}^{c}=\frac{\rho }{\mathrm{\alpha C}}\cdot (\mu_{c}-\mu_{G})$$ (42)

This implies that the last layer parameters *V_L_* are a scaled version of the centered class-wise feature matrix *M* = […*μ_c_* − *μ_G_*…]. Thus, at equilibrium, with quasi-interpolation of the training labels, we obtain $\frac{V_{L}^{⊤}}{∥V_{L}{∥}_{F}}=\frac{M}{{\left.∥M\right|}_{F}}$.

From the SGD equations, we can also see that at equilibrium, with quasi-interpolation, all classifier vectors in the last layer ($V_{L}^{c}$ and, hence, *μ_c_* − *μ_G_*) have the same norm:

$$\begin{array}{c} ∥V_{L}^{c}{∥}_{2}^{2}=\frac{\frac{1}{\mathrm{NC}}\sum_{c\prime =1}^{C}\sum_{n=1}^{N}\left(\rho f_{V}^{\left(c\right)}\left(x_{c\prime n}\right)-{\tilde{e}}_{c\prime }^{\left(c\right)}\right)\rho f_{V}^{\left(c\right)}\left(x_{c\prime n}\right)}{\frac{1}{\mathrm{NC}}\sum_{c\prime =1}^{C}\sum_{n=1}^{N}\langle \rho f_{V}\left(x_{c\prime n}\right)-{\tilde{e}}_{c\prime },\rho f_{V}\left(x_{c\prime n}\right)\rangle }\\ =\frac{\frac{\alpha \left(\alpha -1\right)}{C}+\frac{\alpha \left(\alpha -1\right)}{C\left(C-1\right)}}{\alpha \left(\alpha -1\right)\times \frac{C}{C-1}}=\frac{1}{C} \end{array}$$ (43)

From the quasi-interpolation of the correct class label, we have that $\langle V_{L}^{c},\mu_{c}\rangle =\frac{\alpha }{\rho }$, which means $\langle V_{L}^{c},\mu_{G}\rangle +\langle V_{L}^{c},\mu_{c}-\mu_{G}\rangle =\frac{\alpha }{\rho }$. Now using Eq. 42

$$\begin{array}{c} \langle V_{L}^{c},\mu_{G}\rangle =\frac{\alpha }{\rho }-\frac{\mathrm{\alpha C}}{\rho }∥V_{L}^{c}{∥}_{2}^{2}\\ =\frac{\alpha }{\rho }-\frac{\mathrm{\alpha C}}{\rho }\times \frac{1}{C}=0 \end{array}$$ (44)

From the quasi-interpolation of the incorrect class labels, we have that $\langle V_{L}^{c},\mu_{c\prime }\rangle =\frac{-\alpha }{\rho \left(C-1\right)}$, which means $\langle V_{L}^{c},\mu_{c\prime }-\mu_{G}\rangle +\langle V_{L}^{c},\mu_{G}\rangle =\frac{-\alpha }{\rho \left(C-1\right)}$. Plugging in the previous result and using Eq. 43 yields

$$\begin{array}{cc} \frac{\mathrm{\alpha C}}{\rho }\times \langle V_{L}^{c},V_{L}^{c\prime }\rangle & =\frac{-\alpha }{\rho \left(C-1\right)}\\ ⟹\langle {\tilde{V}}_{L}^{c},{\tilde{V}}_{L}^{c\prime }\rangle & =\frac{1}{∥V_{L}^{c}{∥}_{2}^{2}}\times \frac{-1}{C\left(C-1\right)}=-\frac{1}{C-1} \end{array}$$ (45)

Here, ${\tilde{V}}_{L}^{c}=\frac{V_{L}^{c}}{∥V_{L}^{c}{∥}_{2}}$, and we use the fact that all the norms $∥V_{L}^{c}{∥}_{2}$ are equal. This completes the proof that the normalized classifier parameters form an ETF. Moreover, since $V_{L}^{c}\propto \mu_{c}-\mu_{G}$ and all the proportionality constants are independent of *c*, we obtain $\sum_{c}V_{L}^{c}=0$. This completes the proof of the NC2 condition. NC4 follows then from NC1 to NC2, as shown by theorems in [12].

#### Remarks

• The analyses of the loss landscape and the qualitative dynamics under the square loss in the Qualitative dynamics and Landscape of the empirical risk sections imply that all quasi-interpolating solutions with *ρ* ≥ *ρ*_0_ and *λ* > 0 that satisfy assumption 2 yield NC and have its 4 properties.

• SGD is a necessary requirement in our proof of NC1.

• Our analysis implies that there is no direct relation between NC and generalization. In fact, a careful look at our derivation suggests that NC1 to NC4 should take place for any quasi-interpolating solutions (in the square loss case), including solutions that do not have a large margin. In particular, our analysis predicts NC for datasets with fully random labels—a prediction that has been experimentally verified.

## SGD Bias toward Low-Rank Weight Matrices and Intrinsic SGD Noise

In the previous sections, we assumed that *ρ* and *V_k_* are trained using GF. In this section, we consider a slightly different setting where SGD is applied instead of GF. Specifically, *V_k_* and *ρ* are first initialized and then iteratively updated simultaneously in the following manner

$$\begin{array}{c} \rho \leftarrow \rho -\eta \frac{\partial L_{S\prime }\left(\rho ,{\left\{V_{k}\right\}}_{k=1}^{L}\right)}{\partial \rho }=\rho -\eta \frac{2}{B}\sum_{\left(x_{n},,,y_{n}\right)\in S\prime }\left(1-\rho {\bar{f}}_{n}\right){\bar{f}}_{n}-2\eta \lambda \rho \\ V_{k}\leftarrow V_{k}-\frac{\partial L_{S\prime }\left(\rho ,{\left\{V_{k}\right\}}_{k=1}^{L}\right)}{\partial V_{k}}=V_{k}-\eta \frac{2}{B}\sum_{\left(x_{n},y_{n}\right)\in S\prime }\left(1-\rho {\bar{f}}_{n}\right)\rho \frac{\partial {\bar{f}}_{n}}{\partial V_{k}}-2\eta \nu_{k}V_{k} \end{array}$$ (46)

where $S^{\prime }$ is selected uniformly as a subset of S of size *B*, *η* > 0 is the learning rate, and *ν_k_* is computed according to Eq. 4 with S replaced by $S^{\prime }$.

### Low-rank bias

An intriguing argument for low-rank weight matrices is the following observation that follows from Eq. 5 (see also [7]). The Lemma 8 shows that, in practice, SGD cannot achieve zero gradient for all the minibatches of size smaller than *N*, because, otherwise, all the weight matrices would have very low rank that is incompatible, for generic datasets, with quasi-interpolation.

Lemma 8. Let *f_W_* be a neural network. Assume that we iteratively train ρ and ${\left\{V_{k}\right\}}_{k=1}^{L}$ using the process described above with WD λ > 0. Suppose that training converges, that is $\frac{\partial L_{S\prime }\left(\rho ,{\left\{V_{k}\right\}}_{k=1}^{L}\right)}{\partial \rho }=0$ and $\forall k\in \left[L\right]:\frac{\partial L_{S\prime }\left(\rho ,{\left\{V_{k}\right\}}_{k=1}^{L}\right)}{\partial V_{k}}=0$ for all minibatches $S^{\prime }\subset S$ of size B<∣S∣. Assume that $\forall n\in \left[N\right]:{\bar{f}}_{n}\neq 0$. Then, the ranks of the matrices *V_k_* are at most ≤ 2.

*Proof*. Let *f_V_*(*x*) = *V_L_σ*(*V*_*L* − 1_…*σ*(*V*_1_*x*)…) be the normalized neural network, where *V_l_* ∈ *ℝ*^*d*_*l* + 1_ × *d_l_*^ and ∥*V_l_* ∥ = 1 for all *l* ∈ [*L*]. We would like to show that the matrix $\frac{\partial f_{V}\left(x\right)}{\partial V_{k}}$ is of rank ≤1. We note that for any given vector *z* ∈ *ℝ^d^*, we have *σ*(*v*) = diag (*σ*^′^(*v*)) · *v* (where *σ* is the ReLU activation function). Therefore, for any input vector *x* ∈ *ℝ^n^*, the output of *f_V_* can be written as follows,

$$\begin{array}{cc} f_{V}\left(x\right) & =V_{L}\sigma \left(V_{L-1}\ldots \sigma \left(V_{1}x\right)\ldots \right)\\ & =V_{L}\cdot D_{L-1}\left(x;V\right)\cdots D_{1}\left(x;V\right)\cdot V_{1}\cdot x \end{array}$$ (47)

where *D_l_*(*x*; *V*) = diag [*σ*^′^(*u_l_*(*x*; *V*)))] and *u_l_*(*x*; *V*) = *V_l_σ*(*V*_*l* − 1_…*σ*(*V*_1_*x*)…). We denote *u*_*l*, *i*_(*x*; *V*) as the *i*th coordinate of the vector *u_l_*(*x*; *V*). We note that *u_l_*(*x*; *V*) are continuous functions of *V*. Therefore, assuming that none of the coordinates *u*_*l*, *i*_(*x*; *V*) are zero, there exists a sufficiently small ball around *V* for which *u*_*l*, *i*_(*x*; *V*) does not change its sign. Hence, within this ball, *σ*^′^(*u*_*l*, *i*_(*x*; *V*)) is constant. We define sets $V≔\left\{V|\forall l\leq L:∥V_{l}∥=1\right\}$ and $V_{l,i}=\left\{V\in V|u_{l,i}\left(x;V\right)=0\right\}$. We note that as long as *x* ≠ 0, the set $V_{l,i}$ is negligible within V. Since there is a finite set of indices *l*, *i*, the set ${⋃}_{l,i}V_{l,i}$ is also negligible within V.

Let *V* be a set of matrices for which none of the coordinates *u*_*l*, *i*_(*x*; *V*) are zero. Then, the matrices ${\left\{D_{l}\left(x;V\right)\right\}}_{l=1}^{L-1}$ are constant in the neighborhood of *V*, and therefore, their derivative with respect to *V_k_* are zero. Let *a*^⊤^ = *V_L_* · *D*_*L* − 1_(*x*; *V*)*V*_*L* − 1_⋯*V*_*k* + 1_*D_k_*(*x*; *V*) and *b* = *D*_*k* − 1_(*x*) · *V*_*k* − 1_⋯*V*_1_*x*. We can write *f_V_*(*x*) = *a*(*x*; *V*)^⊤^ · *V_k_* · *b*(*x*; *V*). Since the derivatives of *a*(*x*; *V*) and *b*(*x*; *V*) with respect to *V_k_* are zero, by applying $\frac{\partial a^{⊤}\mathrm{Xb}}{X}=ab^{⊤}$, we have $\frac{\partial f_{V}\left(x\right)}{\partial V_{k}}=a\left(x;V\right)\cdot b{\left(x;V\right)}^{⊤}$ that is a matrix of rank at most 1. Therefore, $\frac{\partial {\bar{f}}_{n}}{\partial V_{k}}=y_{n}\frac{\partial f_{V}\left(x_{n}\right)}{\partial V_{k}}$ is a matrix of rank at most 1. Therefore, for any input *x_n_* ≠ 0, with measure 1, $\frac{\partial {\bar{f}}_{n}}{\partial V_{k}}$ is a matrix of rank at most 1.

Since $\forall k\in \left[L\right]:\frac{\partial L_{S\prime }\left(\rho ,{\left\{V_{k}\right\}}_{k=1}^{L}\right)}{\partial V_{k}}=0$ for all minibatches $S^{\prime }={\left\{\left(x_{i_{j}},y_{i_{j}}\right)\right\}}_{j=1}^{B}\subset S$ of size B<∣S∣, we have

$$\frac{\partial L_{S\prime }\left(\rho ,,,{\left\{V_{k}\right\}}_{k=1}^{L}\right)}{\partial V_{k}}=\frac{2}{B}\rho \sum_{j=1}^{B}\left[\left(1-\rho {\bar{f}}_{i_{j}}\right)\left(-V_{k}{\bar{f}}_{i_{j}}+\frac{\partial {\bar{f}}_{i_{j}}}{\partial V_{k}}\right)\right]=0$$ (48)

Since interpolation is impossible when training with *λ* > 0, there exists at least one *n* ∈ [*N*] for which $\rho {\bar{f}}_{n}\neq 1$. We consider 2 batches $S_{i}^{\prime }$ and $S_{j}^{\prime }$ of size *B* that differ by sample, (*x_i_*, *y_i_*) and (*x_j_*, *y_j_*). We have

$$\begin{array}{cc} \forall i,j\in \left[N\right]:0 & =\frac{\partial L_{S\prime_{i}}\left(\rho ,{\left\{V_{k}\right\}}_{k=1}^{L}\right)}{\partial V_{k}}-\frac{\partial L_{S\prime_{j}}\left(\rho ,{\left\{V_{k}\right\}}_{k=1}^{L}\right)}{\partial V_{k}}\\ & =\frac{2}{B}\cdot \rho \left[\left(1-\rho {\bar{f}}_{i}\right)\left(-V_{k}{\bar{f}}_{i}+\frac{\partial {\bar{f}}_{i}}{\partial V_{k}}\right)-\left(1-\rho {\bar{f}}_{j}\right)\left(-V_{k}{\bar{f}}_{j}+\frac{\partial {\bar{f}}_{j}}{\partial V_{k}}\right)\right] \end{array}$$ (49)

Assume that there exists a pair *i*, *j* ∈ [*N*] for which $\left(1-\rho {\bar{f}}_{i}\right){\bar{f}}_{i}\neq \left(1-\rho {\bar{f}}_{j}\right){\bar{f}}_{j}$. Then, we can write

$$V_{k}=\frac{\left[\left(1-\rho {\bar{f}}_{i}\right)\cdot \frac{\partial {\bar{f}}_{i}}{\partial V_{k}}+\left(1-\rho {\bar{f}}_{j}\right)\cdot \frac{\partial {\bar{f}}_{j}}{\partial V_{k}}\right]}{\left[\left(1-\rho {\bar{f}}_{i}\right){\bar{f}}_{i}-\left(1-\rho {\bar{f}}_{j}\right){\bar{f}}_{j}\right]}$$ (50)

Since $\frac{\partial {\bar{f}}_{i}}{\partial V_{k}}$ and $\frac{\partial {\bar{f}}_{j}}{\partial V_{k}}$ are matrices of rank ≤1 (see the analysis above), we obtain that *V_k_* is of rank ≤2. Otherwise, assume that for all pairs *i*, *j* ∈ [*N*], we have $\alpha =\left(1-\rho {\bar{f}}_{i}\right){\bar{f}}_{i}=\left(1-\rho {\bar{f}}_{j}\right){\bar{f}}_{j}$. In this case, we obtain that for all *i*, *j* ∈ [*N*], we have

$$\left(1-\rho {\bar{f}}_{i}\right)\cdot \frac{\partial {\bar{f}}_{i}}{\partial V_{k}}=\left(1-\rho {\bar{f}}_{j}\right)\cdot \frac{\partial {\bar{f}}_{j}}{\partial V_{k}}=U$$ (51)

Therefore, since $\alpha =\left(1-\rho {\bar{f}}_{i}\right){\bar{f}}_{i}=\left(1-\rho {\bar{f}}_{j}\right){\bar{f}}_{j}$, by Eq. 48,

$$0=\frac{2}{B}\rho \sum_{j=1}^{B}\left[\left(1-\rho {\bar{f}}_{i_{j}}\right)\left(-V_{k}{\bar{f}}_{i_{j}}+\frac{\partial {\bar{f}}_{i_{j}}}{\partial V_{k}}\right)\right]=-2\rho \alpha V_{k}+2\mathrm{\rho U}$$ (52)

Since the network cannot perfectly fit the dataset when trained with *λ* > 0, we obtain that there exists *i* ∈ [*N*] for which $\left(1-\rho {\bar{f}}_{i}\right)\neq 0$. Since ${\bar{f}}_{i}\neq 0$ for all *i* ∈ [*N*], this implies that *α* ≠ 0. We conclude that *V_k_* is proportional to *U*, which is of rank ≤1.

All GD methods try to converge to points in parameter space that have zero or very small gradient; in other words, they try to minimize $∥{\overset{\cdot }{V}}_{k}∥,\forall k$. Assuming separability, $\ell_{n}=\left(1-\rho {\bar{f}}_{n}\right)>0,\forall n$. Equation 10 then implies

$$∥{\overset{\cdot }{V}}_{k}∥=\frac{2\rho }{N}\sum_{n\in B}\ell_{n}∥\frac{\partial {\bar{f}}_{n}}{\partial V_{k}}-f_{n}V_{k}∥$$ (53)

which predicts that the norm of the SGD updates at layer *k* should reflect, asymptotically, the rank of *V_k_*.

#### Is low-rank bias related to generalization?

An obvious question is whether a deep ReLU network that fits the data generalizes better than another one if the rank of its weight matrices is lower. The following result is stated in [8]:

Theorem 7. Let *f_V_ be a normalized neural network, trained with SGD under square loss in the presence of WN. Assume that the weight matrix V_k_* of dimensionality (*n*, *n*) has rank *r* < *n.* Then, its contribution to the Rademacher complexity of the network will be $\sqrt{\frac{r}{n}}$ (instead of 1 as in the typical bound).

### Origin of SGD noise

**Fig. 10.**
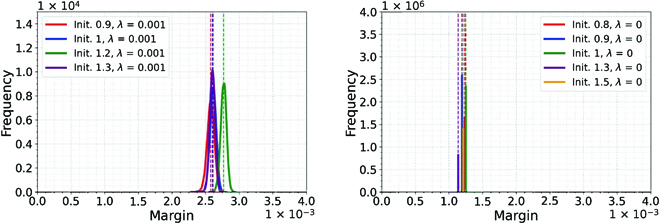
Training margins computed over 10 runs for binary classification on CIFAR10 trained with square loss, LM normalization, and WD *λ* = 0.001 (left) and without WD (right, *λ* = 0) for different initializations (init. = 0.8, 0.9, 1, 1.2, 1.3, and 1.5) with SGD and minibatch size of 128. The margin distribution is Gaussian-like with standard deviation ≈10^−4^ over the training set (*N* = 10^4^). The margins without WD result in a range of smaller margin values, each with essentially zero variance. As mentioned in the text, the norms of the convolutional layers are just the norm of the filters.

Lemma 8 shows that there cannot be convergence to a unique set of weights ${\left\{V_{k}\right\}}_{k=1}^{L}$ that satisfy equilibrium for all minibatches. More details of the argument are illustrated in [54,55]. When *λ* = 0, interpolation of all data points is expected: In this case, the GD equilibrium can be reached without any constraint on the weights. This is also the situation in which SGD noise is expected to essentially disappear: Compare the histograms on the left and the right hand side of Fig. 10. Thus, during training, the solution ${\left\{V_{k}\right\}}_{k=1}^{L}$ is not the same for all samples: There is no convergence to a unique solution but instead fluctuations between solutions during training. The absence of convergence to a unique solution is not surprising for SGD when the landscape is not convex.

## Summary

### The dynamics of GF

In this paper, we have considered a model of the dynamics of, first, GF, and then stochastic GD in overparameterized ReLU neural networks trained for square loss minimization. Under the assumption of convergence to zero loss minima, we have shown that solutions have a bias toward small *ρ*, defined as the product of the Frobenius norms of each layer’s (unnormalized) weight matrix. We assume that during training, there is normalization using an LM of each layer weight matrix but the last one, together with WD with the regularization parameter *λ*. Without WD, the best solution would be the interpolating solution with minimum *ρ* that may be achieved with appropriate initial conditions that are appropriate.

#### Remarks

• The bias toward small *ρ* solutions induced by regularization with *λ* > 0 may be replaced—when *λ* = 0—by an implicit bias induced by small initialization. With appropriate parameter values, small initialization allows convergence to the first quasi-interpolating solution for increasing *ρ* from ≈ 0 to *ρ*_0_. For *λ* = 0, we have empirically observed solutions with large *ρ* that are suboptimal and probably similar to the NTK regime.

• A puzzle that remains open is why BN leads to better solutions than LN and WN, despite similarities between them. WN is easier to formalize mathematically as LN, which is the main reason for the role it plays in this paper.

### Generalization and bounds

Building on our analysis of the dynamics of *ρ*, we derive new norm-based generalization bounds for CNNs for the special case of nonoverlapping convolutional patches. These bounds show (a) that generalization for CNNs can be orders of magnitude better than for dense networks and (b) that these bounds can be empirically loose but nonvacuous despite overparametrization.

#### Remarks

• For *λ* > 0, a main property of the minimizers that upper bounds their expected error is *ρ*, which is the inverse of the margin: We prove that among all the quasi-interpolating solutions, the ones associated with smaller *ρ* have better bounds on the expected classification error.

• The situation here is somewhat similar to the linear case: For overparameterized networks, the best solution in terms of generalization is the minimum norm solution toward which GD is biased.

• Large margin is usually associated with good generalization [56]; in the meantime, however, it is also broadly recognized that margin alone does not fully account for generalization in deep nets [28,31,57]. Margin, in fact, provides an upper bound on generalization error, as shown in Generalization: Rademacher Complexity of Convolutional Layers. Larger margin gives a better upper bound on the generalization error for the same network trained on the same data. We have empirically verified this property by varying the margin using different degrees of random labels in a binary classification task. While training gives perfect classification and zero square loss, the margin on the training set together with the test error decreases with the increase in the percentage of random labels. Of course, large margin in our theoretical analysis is associated with regularization that helps minimizing *ρ*. Since *ρ* is the product of the Frobenius norm, its minimization is directly related to minimizing a Bayes prior [58], which is itself directly related to minimum description length principles.

• We do not believe that flat minima directly affect generalization. As we described in the Interpolation and quasi-interpolation section, degenerate minima correspond to solutions that have zero empirical loss (for *λ* = 0). Minimizing the empirical loss is a (almost) necessary condition for good generalization. It is not, however, sufficient since minimization of the expected error also requires a solution with low complexity.

• The upper bound given in Generalization: Rademacher Complexity of Convolutional Layers, however, does not explain by itself details of the generalization behavior that we observe for different initializations (see Fig. 4), where small differences in margin are actually anticorrelated with small differences in test error. We conjecture that margin (related to *ρ*) together with sparsity of F may be sufficient to explain generalization.

### Neural collapse

Another consequence of our analysis is a proof of NC for deep networks trained with square loss in the binary classification case without any assumption. In particular, we prove that training the network using SGD with WD, induces a bias toward low-rank weight matrices and yields SGD noise in the weight matrices and in the margins, which makes exact convergence impossible, even asymptotically.

#### Remarks

• A natural question is whether NC is related to solutions with good generalization. Our analysis suggests that this is not the case, at least not directly: NC is a property of the dynamics, independently of the size of the margin that provides an upper bound on the expected error. In fact, our prediction of NC for randomly labeled CIFAR10 was confirmed originally in then preliminary experiments by our collaborators (Papyan et al. [12]) and more recently in other papers (see for instance, [33]).

• Margins, however, do converge to each other but only within a small *ϵ*, implying that the first condition for NC [12] is satisfied only in this approximate sense. This is equivalent to saying that that SGD does not converge to a unique solution that corresponds to zero gradient for all data point.

## Conclusion

Finally, we would like to emphasize that the analysis of this paper supports the idea that the advantage of deep networks relative to other standard classifiers is greater for the problems to which sparse architectures such as CNNs can be applied. The reason is that CNNs reflect the function graph of target functions that are compositionally sparse and, thus, can be approximated well by sparse networks without incurring in the curse of dimensionality. Despite overparametrization, the compositionally sparse networks can then show good generalization.

## Data Availability

The experimental dataset we used in this paper is a public CIFAR10 dataset, and it can be accessed and downloaded from https://www.cs.toronto.edu/~kriz/cifar.html.
